# Insights on the Atmospheric-Pressure Plasma-Induced Free-Radical Polymerization of Allyl Ether Cyclic Carbonate Liquid Layers

**DOI:** 10.3390/polym13172856

**Published:** 2021-08-25

**Authors:** Edyta M. Niemczyk, Alvaro Gomez-Lopez, Jean R. N. Haler, Gilles Frache, Haritz Sardon, Robert Quintana

**Affiliations:** 1 Department of Materials Research and Technology, Luxembourg Institute of Science and Technology (LIST), 4422 Belvaux, Luxembourg; edyta.niemczyk@list.lu (E.M.N.); jean.haler@list.lu (J.R.N.H.); gilles.frache@list.lu (G.F.); 2 Department of Physics and Materials Science, University of Luxembourg, 4365 Esch-sur-Alzette, Luxembourg; 3 POLYMAT and Polymer Science and Technology Department, Faculty of Chemistry, University of the Basque Country UPV/EHU, 20018 Donostia-San Sebastián, Spain; alvaro.gomez@ehu.eus (A.G.-L.); haritz.sardon@ehu.eus (H.S.)

**Keywords:** allyl-substituted cyclic carbonate, free-radical polymerization, atmospheric-pressure plasma

## Abstract

Plasma-induced free-radical polymerizations rely on the formation of radical species to initiate polymerization, leading to some extent of monomer fragmentation. In this work, the plasma-induced polymerization of an allyl ether-substituted six-membered cyclic carbonate (A6CC) is demonstrated and emphasizes the retention of the cyclic carbonate moieties. Taking advantage of the low polymerization tendency of allyl monomers, the characterization of the oligomeric species is studied to obtain insights into the effect of plasma exposure on inducing free-radical polymerization. In less than 5 min of plasma exposure, a monomer conversion close to 90% is obtained. The molecular analysis of the oligomers by gel permeation chromatography coupled with high-resolution mass spectrometry (GPC-HRMS) further confirms the high preservation of the cyclic structure and, based on the detected end groups, points to hydrogen abstraction as the main contributor to the initiation and termination of polymer chain growth. These results demonstrate that the elaboration of surfaces functionalized with cyclic carbonates could be readily elaborated by atmospheric-pressure plasmas, for instance, by copolymerization.

## 1. Introduction

The synthesis and direct deposition of polymeric thin films by atmospheric-pressure plasma-induced polymerization is an appealing approach for the elaboration of functional surfaces [1]. A major contribution to its success is owed to its fast deposition rates, the good retention of the chemical structure of the monomers in the deposited film and the use of solvent-less, mild reaction conditions, usually at room temperature. Additionally, working at atmospheric pressure eases the scalability of the deposition process [2] and allows the use of precursors in the liquid phase [3]. This extends the application to monomers of very low vapor pressure, which are not suitable for processing in the vapor phase. Vinyl functional monomers are readily polymerized by plasma-induced free-radical polymerization leading to the deposition of thin films with a large variety of functional groups, including cyclic groups, such as epoxide [4,5], lactam [6] and catechol/quinone [7]. These groups are of interest because they can improve adhesion between the thin film and the treated surface. Their reactivity also allows post-polymerization modifications of the surface, for instance, for biomolecule immobilization for practical applications in biomedical [7] and environmental protection fields [4]. Despite the advantages shown by atmospheric-pressure plasma-induced free-radical polymerization in the preservation of the chemical structure of monomers, monomer fragmentation should occur in some extent to initiate polymerization. This can reduce the amount of cyclic functional groups in the resulting polymer. The retention of epoxide groups, by far the most studied case, has been found to be dependent on the nature of the polymerizable group, i.e., vinyl or allyl. Manakhov et al. reported that the concentration of the epoxide groups in the plasma-polymerized polymer was lower when allyl glycidyl ether (AGE) was used instead of glycidyl methacrylate (GMA) [8]. In addition to the lower retention of the epoxide groups, the polymerization of AGE led to lower deposition rates. This difference could be attributed to the well-known difficulty of allyl monomers to polymerize and the formation of oligomers or medium-molecular-weight polymers [9,10]. Nevertheless, the free-radical polymerization of allyls, either by conventional wet chemistry or by plasma polymerization [11,12,13,14], has received less attention than that of their vinyl counterparts.

Cyclic carbonates are gaining attention as sustainable compounds in the context of green and sustainable chemistry. Their synthesis can involve the use of carbon dioxide as a building block, can be applied as green solvents and can substitute toxic reactants currently employed in the chemical industry [15]. Among the different cyclic carbonate sizes, five-membered cyclic carbonates are easily prepared through [3 + 2] CO_2_ insertion into their corresponding (bio-based) epoxy precursors [16]. Nonetheless, their low reactivity restricts their use in some applications. Six-membered cyclic carbonates present greater reactivity at room temperature and, additionally, higher stability than seven- and eight-membered cyclic carbonates, which can be crucial to retain their structure under plasma conditions [17]. Moreover, recently, a one-step synthesis procedure has been reported using 1,3-diols and CO_2_ at atmospheric pressure and ambient temperature to prepare six-membered cyclic carbonates, increasing the interest for these monomers as green and sustainable reactants [18].

The introduction of pendent groups in cyclic carbonates increases their reactivity and allows the production of functionalized polycarbonates by ring-opening polymerization (ROP). Several functionalized cyclic carbonates have already been explored, such as allyl, alkyne and maleimide. Allyl pendent groups have been considered as the intermediate step for the post-functionalization of polycarbonates by a thiolene coupling reaction [19] and to increase their mechanical stability by free-radical cross-linking [20,21]. Interestingly, recent studies have shown that allyl ether-substituted six-membered cyclic carbonates can be obtained as a value-added byproduct of the upcycling of mixed plastic waste [22], in addition to ring-closing depolymerization [23]. Otherwise, polymers decorated with cyclic carbonates have gained interest in the production of thermoset plastics and coatings [24]. In the latter case, the reaction of the carbonates with amines is exploited to promote the adhesion of the coating on polyurethane-based materials. The reactivity with amines has also been explored for the coupling reaction of biomolecules [25].

The synthesis of polymers with pendent cyclic carbonates by plasma processes has not yet been reported. In comparison with the epoxide group, cyclic carbonates are notably less reactive [24] and might be more stable to plasma exposure. In this work, the plasma-induced polymerization of an allyl ether-substituted six-membered cyclic carbonate (A6CC, 5-((allyloxy)methyl)-5-ethyl-1,3-dioxan-2-one) was studied using a cold, atmospheric-pressure dielectric barrier discharge (DBD) plasma. In particular, the monomer A6CC was exposed to the plasma in the form of a liquid layer due to its very low vapor pressure. After reiterated plasma exposure, both the conversion of the allyl group and the retention of the cyclic carbonate were investigated by FTIR and NMR spectroscopies. High-resolution mass spectrometry (HRMS) analyses of the, as expected, low-molecular-weight products was performed to identify the chemical formula of the products to obtain insights into the plasma-induced polymerization mechanism.

## 2. Materials and Methods

### 2.1. Synthesis of 5-((allyloxy)methyl)-5-ethyl-1,3-dioxan-2-one: A6CC

The synthesis of 5-((allyloxy)methyl)-5-ethyl-1,3-dioxan-2-one was carried out as described elsewhere [17]. Ethyl chloroformate (6.81 g, 62.75 mmol, 97%, Sigma-Aldrich, Madrid, Spain) was added dropwise to a solution of trimethylolpropane allyl ether (TMPAE) (5.44 g, 31.24 mmol, Sigma-Aldrich, 90%) and triethylamine (6.96 g, 68.73 mmol, Sigma-Aldrich, 99.5%) in 200 mL of dried THF at 0 °C over a period of 40 min. The reaction mixture was then stirred at room temperature for 2 h. The precipitated triethylamine hydrochloride was filtrated off, and the filtrate was concentrated under vacuum. Then, the crude was diluted with ethyl acetate (300 mL) and washed two times with aqueous hydrochloric acid (1 M) and two times with deionized water. The organic phase was dried over anhydrous magnesium sulfate and concentrated under vacuum. The residue was purified by column chromatography (eluent 30/70 ethyl acetate/hexane). The pure product A6CC was obtained as a colorless liquid with 61% yield.

### 2.2. Atmospheric-Pressure Plasma-Induced Polymerization of A6CC

Thin liquid layers of the monomer were formed over silicon substrates using a spin coater (Laurell Technologies, WS-650-23B, North Wales, PA, United States) operating at 10,000 rpm for 30 s. Any solvent was required in this process. The substrate covered with the thin liquid layer was then transferred to an atmospheric-pressure dielectric barrier discharge (DBD) plasma reactor, described elsewhere [7]. Briefly, A SOFTAL 7010R corona generator provided a 10 kHz sinusoidal electrical excitation, and the plasma power was adjusted to deliver between 0.53 and 1.60 W·cm^−2^. The active plasma zone of 18.72 cm^2^ was created between two parallel and horizontal electrodes. The top electrode was formed by two high voltage (HV) bars, covered with a thick alumina-dielectric barrier material, separated by a third bar used to introduce the plasma gas (Ar 99.999%, 20 slm). The grounded electrode was the moving table where the substrates were placed. The speed of the table was kept constant at 50 mm·s^−1^, and the number of runs through the plasma zone were 30, 40, 50, 70 or 90 runs. The effective plasma exposure duration for each table run was 3 s. For the series of samples, the following annotation was used: monomer acronym, dash, the number of table runs under plasma; for instance, A6CC-30 refers to a sample that underwent 30 runs through the plasma zone, with 90 s of effective plasma exposure. The samples were washed out with a solvent to recover the polymer from the silicon surface. For characterization, pooled samples of at least 5 replicates were used.

### 2.3. Characterization

One-dimensional spectra ^1^H NMR and 2-dimensional spectra heteronuclear single quantum coherence (HSQC), heteronuclear multiple bond correlation (HMBC) and bidimensional ^1^H-^1^H correlation spectroscopy (COSY) were acquired on a Bruker Avance-III HD spectrometer (Rheinstetten, Germany) operating at a 1H frequency of 600 MHz. The delay between the scans was 10 s. The following parameters were used: 2048 data points along the f2 dimension, 256 free induction decays in the f1 dimension, a pulse width of 11.05 ms, a spectral width of 3448 Hz (1H) and the number of scans was 16 with a digital resolution of 3.97 Hz per point. Experiments were performed at room temperature. Monomers and plasma-deposited layers were dissolved in deuterated chloroform (CDCl3) at room temperature. Assignments were performed using a combination of COSY, HSQC and HMBC spectra. Chemical shifts are given as d (ppm), and the coupling constants (Hz) were reported as J. Resonances were identified from the literature chemical shift data.

FTIR transmission measurements of the polymer deposited on the silicon wafer were performed on a Bruker Vertex 70 spectrometer (Ettlingen, Germany) equipped with an MCT detector. All FTIR spectra were normalized according to the C=O stretching band at 1755 cm^−1^. The spectra were acquired between 600 and 4000 cm^−1^ with an accumulation of 128 scans and a resolution of 4 cm^−1^. Spectra acquisition was controlled by the OPUS 7.2 software package (Bruker Corporation, Billerica, MA, USA).

A 1260 Infinity II gel permeation chromatograph (GPC, Agilent Technologies, Santa Clara, CA, USA) was used to determine the Mn, Mw and PDI of the polymers. The chromatograph was equipped with an integrated RI detector, a PLgel 5 mm MIXED-C, PLgel 5 mm MIXED-D columns and a PLgel guard column (Agilent Technologies, Santa Clara, CA, USA). Chloroform was used as an eluent with a flow rate of 1.0 mL min^−1^ at 40 °C. Polystyrene standards (Agilent Technologies, Santa Clara, CA, USA, Mp = 162 − 1500 × 103 g mol^−1^) were used to perform calibration.

GPC-HRMS analyses were carried out with the HPLC system (Ultimate 3000, Dionex, Thermo Scientific, Waltham, MA, USA) coupled online to an LTQ/Orbitrap Elite mass spectrometer (Thermo Fisher Scientific, San Jose, CA, USA) with an Ion Max source, equipped with a heated electrospray (H-ESI) probe from Thermo Scientific. Samples were dissolved in chloroform and filtered over a 0.45 μm pore size membrane prior to injection. A mesopore column of 3 μm (300 × 7.5 mm) from Agilent Technologies with an exclusion limit of 25 kg·mol^−1^ was used at 30 °C working in THF. The flow rate was set to 1 mL·min^−1^ and split post-column toward the UV detection on one hand and the ESI-HRMS detection on another hand. Ammonium acetate was added post-split to promote ionization. The spray voltage was set to 3.2 kV. The calculations were carried out based on the PolyCalc web-based assignment tool [26].

## 3. Results

**Figure 1:**
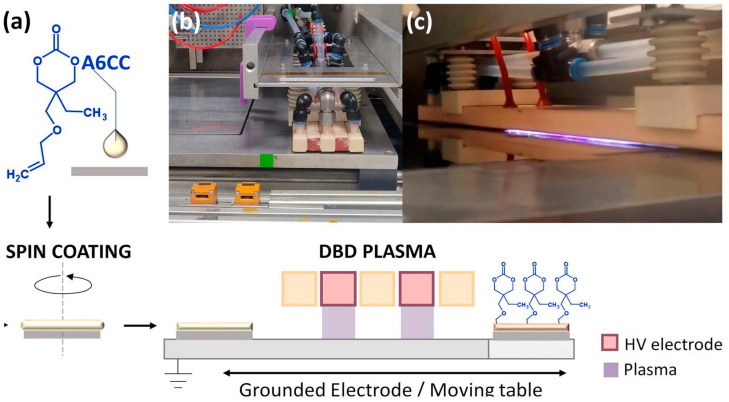
Schematic representation of the process for the atmospheric-pressure plasma-induced polymerization of liquid layers of the allyl ether-substituted cyclic carbonate (A6CC) at room temperature (**a**). Picture of the DBD applicator over the moving table. Plasma gas is supplied in between the two high voltage electrodes (**b**). DBD side view with plasma engaged (**c**).

The chemical structure of the A6CC monomer and the experimental set-up used for the polymerization are shown in Figure 1. The monomer is a clear liquid at room conditions and has a very low vapor pressure, both properties facilitated the formation by the spin coating of very thin liquid layers over the silicon substrates. The values of the plasma power used for the plasma-induced polymerization, i.e., 0.53 and 1.60 W·cm^−2^, were chosen within the most common range of values reported for the plasma polymerization of liquid monomers containing vinyl and allyl groups [7]. The lower value was also the minimal power able to sustain the plasma discharge in the DBD reactor, without modifying the gap between electrodes. After exposition to the plasma, the visual appearance of the initial liquid layer over the silicon surface varied to an oily-like one. When rubbing, the samples showed different degrees of consistency/viscosity, suggesting the formation of products of higher average molecular weights. The samples showing a higher consistency were those obtained using the highest power and longer exposition times. At the lowest power, the liquid-like appearance was always observed. To complement this set of qualitative results and to correlate the effect of the plasma exposure to changes on the chemical structure of the A6CC monomer, Figure S1 shows the FTIR spectra of the monomer and four different samples showing the liquid-like appearance. Considering the bands attributed to allyl groups, i.e., 1648, 993 and 930 cm^−1^ [27,28], after 10 runs, regardless of the plasma power used, a drop in the intensity of these absorption bands was evidenced. Only in the spectrum of the sample polymerized with the highest exposition time and at the highest power were those bands not detectable.

**Figure 2:**
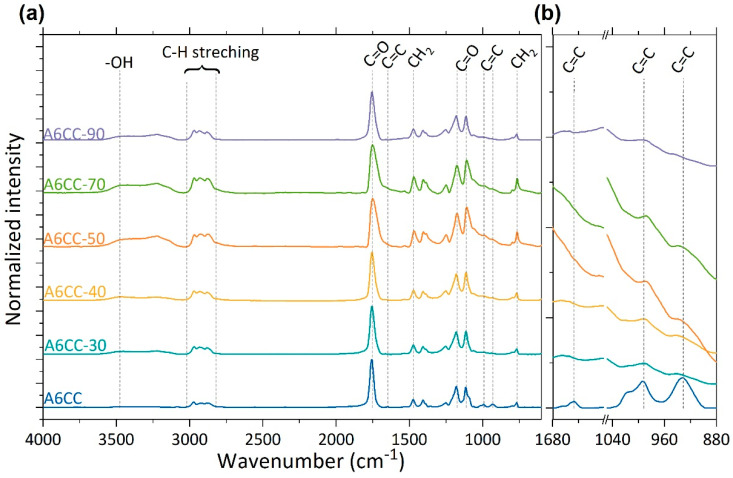
Representative FTIR spectra of the A6CC liquid layers as a function of the number of runs, up to 90, of plasma exposure (power: 1.60 W·cm^−2^). (**a**,**b**) zoom of the bands attributed to the allyl C-C double bond. The spectra were normalized with respect to the peak of the carbonate carbonyl band.

The higher power was then considered in order to carry out a systematic study where the number of runs was increased up to three-fold. Figure 2 shows the recorded FTIR spectra, normalized according to the intensity of the absorption band at 1755 cm^−1^ (C=O stretching carbonate ring), of samples exposed to plasma for 30, 40, 50, 70 and 90 runs. Increasing the exposition time not only led to the decrease in and disappearance of the bands attributed at the allyl groups (Figure 2b), but the formation of alkyl backbone could be detected by the appearance of and increase in the plasma exposition time of the absorption bands in the range of 3050–2800 cm^−1^, corresponding to C-H stretching, and at 760 cm^−1^, related to the rocking vibrations of CH_2_ of the long alkyl chains [8,12,29,30].

**Figure 3:**
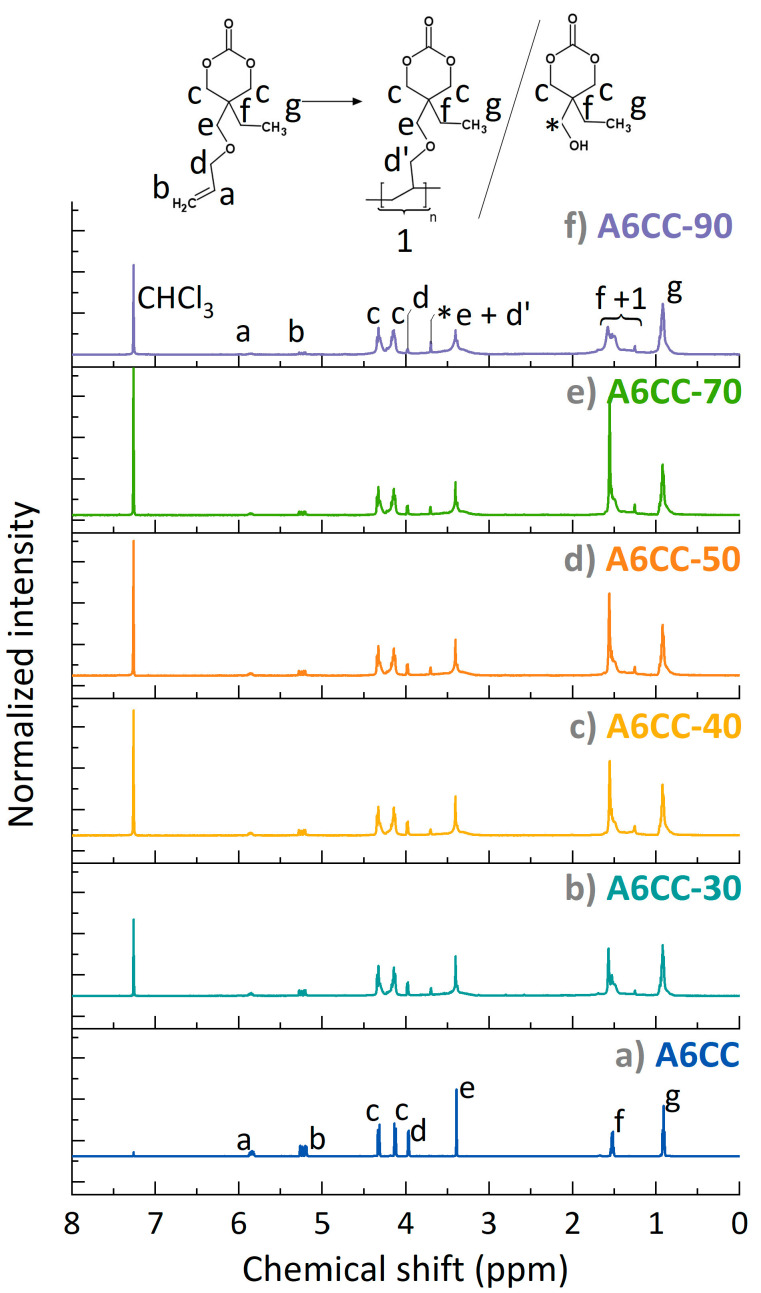
^1^H NMR spectra (in CDCl3) normalized according to the methylene group (signal g) of monomer (**a**) A6CC before and after the plasma-induced polymerization. (**b**) A6CC-30, (**c**) A6CC-40, (**d**) A6CC-50, (**e**) A6CC-70 and (**f**) A6CC-90. * assigned to the chemical structure shown in the figure.

**Figure 4:**
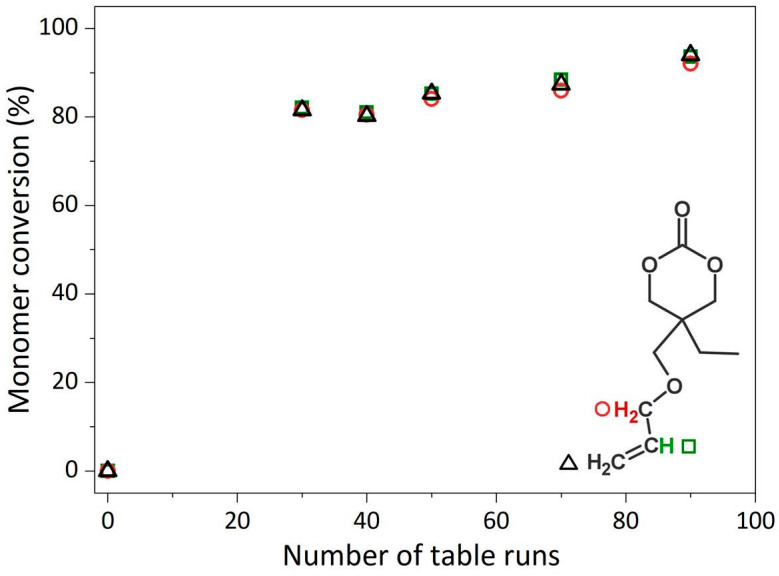
Conversion of the allyl group in function of plasma exposure time (number of table runs) calculated based on the signals from the protons of the allyl group (=CH_2_, triangle and =CH-, square symbols) and the α-carbon (-CH_2_-, circle symbol) in the ^1^H NMR spectra. The integral of the protons of the methyl group were considered as internal reference for the calculation.

The series of samples was also analyzed by NMR spectroscopy (Figure 3). In agreement with the FTIR results, the consumption of the allyl groups was nearly complete as evidenced by the reduction of the signals at 5.85 ppm (a, methine, O-CH_2_-**CH**=CH_2_) and 5.23 (b, methylidene, O-CH_2_-CH=**CH_2_**) already within 30 runs. Considering the protons of the carbon in the alpha position of the allyl group, **d** (O-**CH_2_**-CH=CH_2_), their signal shifted toward a lower chemical shift, from 3.97 to 3.48–3.15 ppm, overlapping with signal **e**, resulting in a broad peak (Figure S2) [31]. Additionally, new peaks (labeled as **1**) at the 1.45–1.70 ppm region appeared. These results evidenced the formation of a polymeric alkyl backbone. Otherwise, the peaks assigned to the ethyl chain pendent from the cycle, **f** methylene group at 1.52 ppm and **g** methyl group at 0.92 ppm, remained at a similar chemical shift along the polymerization process. The connectivity of **f** (1.56 ppm) with **g** (0.94 ppm) could be stablished by the acquisition of two-dimensional COSY NMR spectra (Figure S3). The conversion of the allyl group was determined by comparison with the integral of the band attributed to the protons of the methyl group (labeled as **g**) with the integrals of protons **a** and **b** of the allyl groups. As shown in Figure 4, the consumption of the carbon–carbon double bond reached 82% for A6CC-30 and remained at 94% for the most prolonged plasma exposed sample, A6CC-90 [32]. This approach to determine the conversion did not consider that methyl groups can also be generated as end groups by the free-radical polymerization of the allyl group; therefore, the reported values can be considered as conservative.

While the set of results provided experimental evidence of the step-growth polymerization of the allyl groups, the results were further scrutinized to assess the preservation of the closed structure of the cyclic carbonate. First, in ^1^H-NMR, the close ring structure is characterized by two distinct doublets (**c**) at resonances 4.13 ppm and 4.33 ppm assigned to the diastereotopic protons H_eq_ and H_ax_. In all the recorded spectra, the signals were detected, and their integrals remained similar between them. Indeed, the opening of the ring would result in free-rotating protons, which would appear as a single peak at 4.2 ppm (Figure S4) [33].

**Figure 5:**
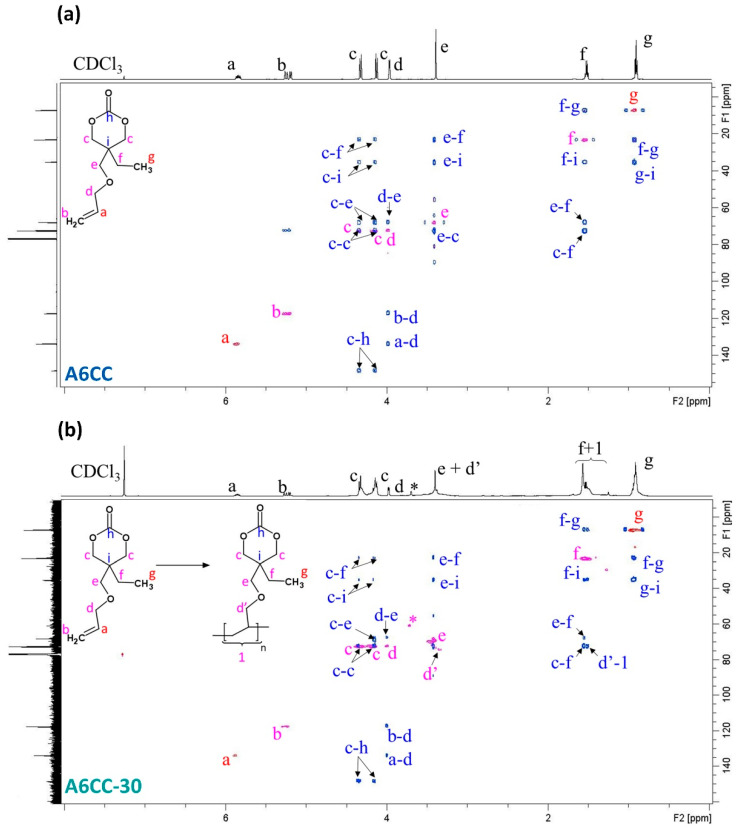
HSQC-HMBC cross-peaks of the (**a**) A6CC and (**b**) A6CC-30 samples, where red (odd) and pink (even) contours represent HSQC, and blue contours correspond to HMBC. HSQC reveals carbon–proton interaction, while HMBC shows the interaction between the proton and neighboring carbons. (*) corresponds to the methylene groups of the detected by-product.

Further confirmation of the closed ring structure was achieved by the acquisition of two-dimensional NMR spectra. COSY NMR (Figure S3) evidenced the correlation of the diastereotopic protons interlocked in the cycle. HSQC and HMBC mutual proton–carbon correlations (Figure 5 and Figure S5) were carried out to confirm the ring structure is connected, as a pendent group, to the formed polymeric chain. As shown in Figure 5a, the closed ring structure of the monomer implies two cross-peaks between diastereotopic protons forming the cyclic carbonate, labeled as **c** (^1^H 4.13 and 4.33 ppm), and carbonate carbon as revealed by 2D HMBC spectra (**c–h**: ^1^H 4.13 and 4.33 ppm/^13^C 148.6 ppm). The quaternary carbon forms 2D HMBC cross-peaks with diastereotopic protons (**c–i**: ^1^H 4.13 and 4.33 ppm/^13^C 35.8 ppm), methoxy (**e–i**: ^1^H 3.39/^13^C 35.8 ppm) and the ethylene group (methylene **f-i**: ^1^H 1.52/^13^C 35.8 ppm and methyl **g–i**: ^1^H 0.92/^13^C 35.8 ppm). The same HMBC cross-peaks pattern could be observed for A6CC-30 (Figure 5b) and A6CC-90 (Figure S5). Similarly, their 2D HSQC spectra revealed that the protons with resonance in the range of 3.30–3.45 ppm corresponded to two different carbons with chemical shifts 68.2 and 72.0 ppm. The latter ^13^C shift, labeled as **d**, resembles the peak signal for the A6CC monomer, confirming that the 2D HSQC cross-peak ^1^H 3.32 ppm–^13^C 72.0 ppm is linked to the methoxy group from the former allyl group. For the polymerized A6CCs, the peak was denoted as **d’** [34]. The signal **d’** formed long-distance decoupling with the signal labeled as **f + 1** (2D HSQC, ^1^H 1.55 ppm ^13^C 72.0 ppm), evidencing the ether link in between the cyclic structure and the polymeric backbone. These results provided clear evidence to confirm the preservation of the pendent cyclic carbonate structure even after long exposures to the plasma. Nevertheless, the presence of new peaks from by-products was also detected. Labeled as (*) (^1^H 3.70 ppm/^13^C 61.00 ppm) could be correlated with protons from methylene groups. COSY-NMR spectra evidenced an interaction between them, and HSQC-HMBC evidenced a correlation of the signal (*) with the closed carbonate cycle. The chemical structure of this compound and its origin are further discussed by mass spectrometry analyses.

**Figure 6:**
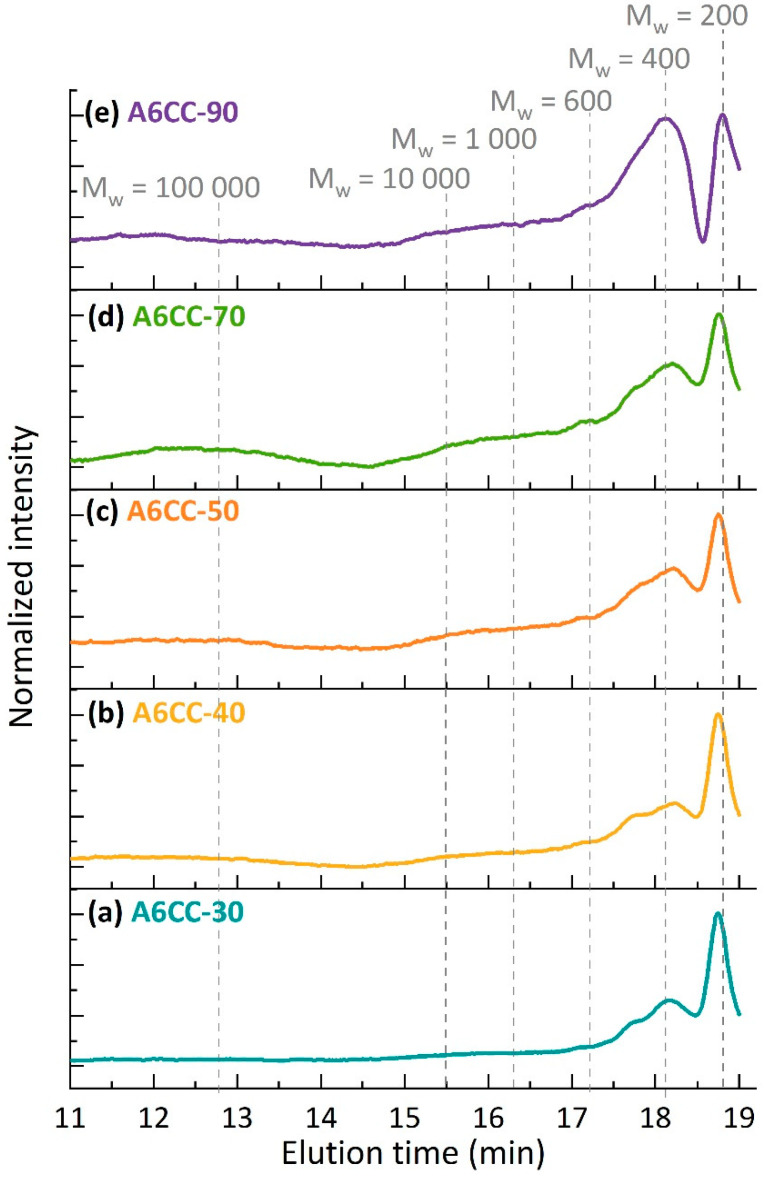
GPC chromatograms of the of the series of A6CC samples in chloroform: (**a**) A6CC-30, (**b**) A6CC-40, (**c**) A6CC-50, (**d**) A6CC-70 and (**e**) A6CC-90. For reference, dashed lines indicate the weight average molecular weights of the polystyrene calibration curve.

The determination of the molecular weights of the deposited products was carried out by GPC. The chromatograms, normalized using the peak with the highest elution time, are shown in Figure 6. They mainly revealed the presence of polydisperse products of low molecular weights, ranging from 200 to 1000 g·mol^−1^. The presence of traces of higher molecular weight polymers could also be inferred from the chromatograms. Increasing the plasma exposure time shifted the molecular weight distribution curve toward high molecular weights, indicating an increase in the monomer conversion. The range of molecular weights obtained agrees with the low degree of polymerization commonly reported on the free-radical polymerization of allyl monomers by conventional wet chemistry methods [10]. In the gas phase, the plasma-induced free-radical polymerization of allyl alcohol and allylamine has also been reported [10,35]. Then, the same trends were observed with the formation of oligomers not higher than 10–20 repeating units [36]. In all these cases, the main mechanism behind this result has been related to the degradative monomer chain transfer.

**Figure 7:**
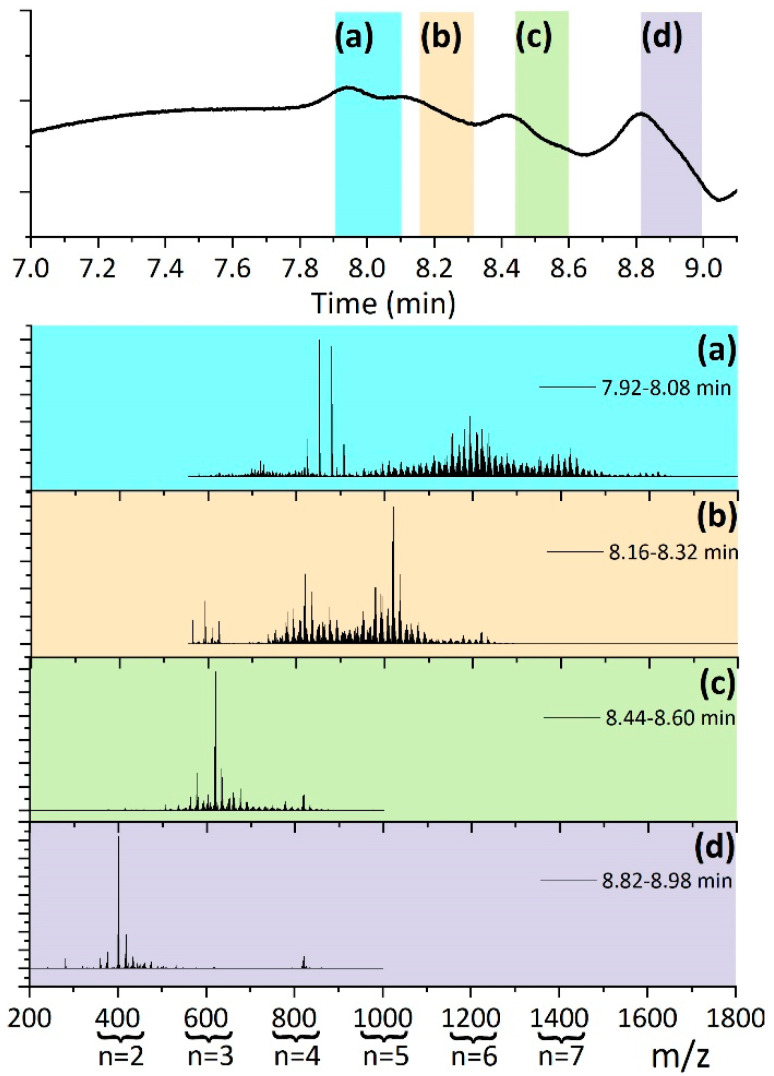
Chromatogram of the A6CC-90 sample obtained by GPC-HRMS. For each elution time range (**a**) 7.92–8.08 min, (**b**) 8.16–8.32 min, (**c**) 8.44–8.60 min and (**d**) 8.82–8.98 min the corresponding HRMS spectrum is displayed. The ranges are indicated by color bands in the GPC chromatogram. For reference, the number of repeating units (n) of the detected oligomers is given.

The low degree of polymerization of the products made it meaningful to attempt their structural identification by gel permeation chromatography (GPC) coupled with high-resolution mass spectrometry (HRMS). The MW distribution of the samples is shown in Figure S6. As an effect of the exclusion limit of the mesopore column used in the GPC-HRMS setup, in all the samples, a peak at 25 kg·mol^−1^ was observed. This confirms the inferred presence of traces of high-molecular-weight polymers. Likewise, the relative intensity of this peak increased with the plasma exposure time. For each sample, the recorded chromatogram with the overlay representation of the HRMS spectra is included in the Supporting Information (Figure S7). As an example, Figure 7 shows the HRMS spectra corresponding to each of the peaks detected in the GPC chromatogram of the sample with the lowest exposition to the plasma, A6CC-30. As a first simple approach, just taking into consideration the *m*/*z* value of ionized adducts of [(A6CC)_n_ + H^+^/NH_4_^+^] oligomers, the number of repeating units reached ca. 7, regardless of the exposure time.

As expected for plasma-induced polymerizations, even with a first fractionation by GPC (Figure S8), the high complexity of the HMRS spectra revealed the presence of a mixture of compounds of different chemical structures. Indeed, in absence of a chemical initiator, as it would be the case for conventional radical polymerizations, plasma-induced polymerizations rely on the creation of radical species to initiate the reaction. Since plasmas are composed of a wide variety of reactive species, including highly energetic species, a plurality of non-specific reactions (i.e., fragmentation or dissociation, ionization, recombination or integration) can take place. These radical species can also terminate polymer chain growth. Recent studies by matrix-assisted laser desorption/ionization high-resolution mass spectroscopy (MALDI-HRMS) on plasma-induced polymerized poly (alkyl acrylates) have demonstrated that the simple hypothesis of a single σ-bond breakdown per molecule to initiate the free-radical polymerization was able to resolve most of the recorded spectra. To show that, the authors combined the mass of the repeating unit (and its multiples) with the masses of up to 25 different radical and neutral fragments resulting from a single bond breakdown, considering them either as the initial and/or final end group [37]. For the sake of example, in Figure S8, the structures of potential radical species are compiled resulting from a single fragmentation of the A6CC monomer by plasma-induced homolytic cleavage.

**Figure 8:**
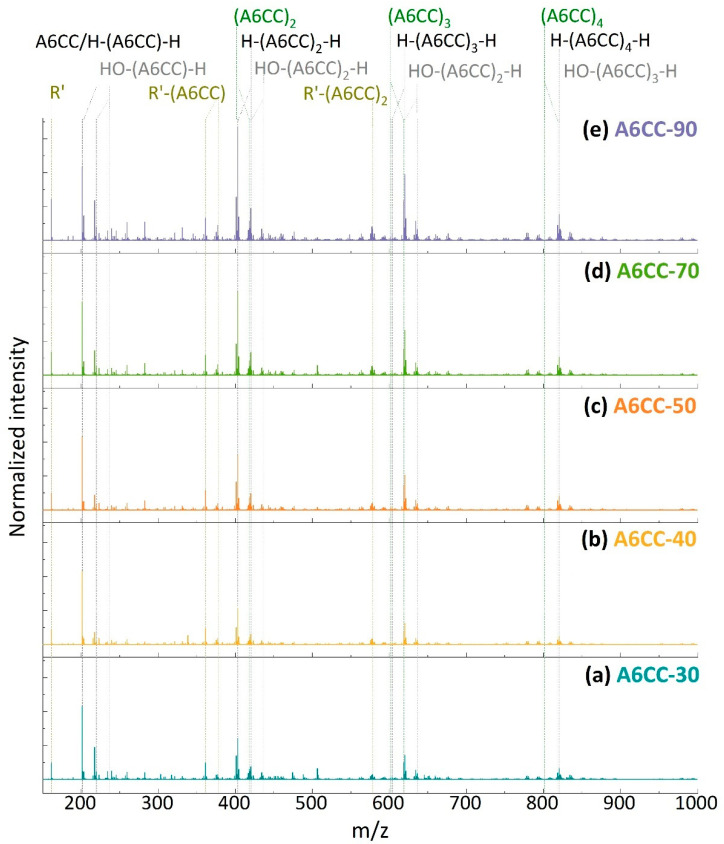
HRMS spectra of plasma-induced polymerization of A6CC in the mass range *m*/*z* = 150–1000 of (**a**) A6CC-30, (**b**) A6CC-40, (**c**) A6CC-50, (**d**) A6CC-70 and (**e**) A6CC-90. The data plotted are the result of the average spectra between 5.5 and 11 min, and the spectra were normalized to the monomer signal [A6CC + H^+^]. The mass peaks resulting from the oligomer formation are indicated by dashed lines, where the color corresponds to the structures shown in Figure 9. The exact *m*/*z* values assigned to these structures are listed in Table S1.

**Figure 9:**
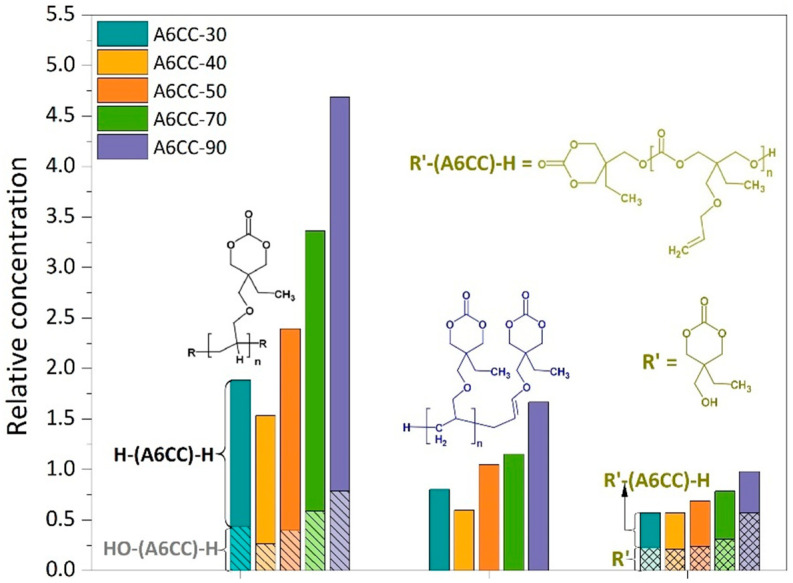
Relative signal intensities to [A6CC + H^+^] monomer adducts of the products formed by plasma-induced polymerization (data from Table S1).

In this study, only the main peaks and isotopic patterns detected in the HRMS spectra were addressed. For each polymerization time, Figure 8 shows the average mass spectra of all the eluted samples, while the *m*/*z* values for the most relevant peaks are summarized in Table S1. In all the spectra, the ionized adducts of the monomer chemical structure (*m*/*z*: 201.113 H^+^ or 218.139 NH_4_^+^) were detected, and the signal was used to normalize the spectra. In spite of all the combinations that were assayed considering the potential fragments from a single σ-bond breakdown and the mass of the A6CC repeating unit, most of the masses detected with higher intensity could be correlated with the ionized adducts of oligomers of different degrees of polymerization (n) with structures [H-(A6CC)_n_-H + H^+^/NH_4_^+^], [H-(A6CC)_n_-CH_2_CH=CHOCH_2_(C_6_H_9_O_3_) + H^+^/NH_4_^+^] and less abundant [H-(A6CC)_n_-OH + H^+^/NH_4_^+^] (as shown in Figure 9). While the unexpected high detection of oligomeric species with hydrogen atoms as the initial end group, thus suggesting the chain growth was initiated by a hydrogen radical, will still need to be properly addressed, the combination of the sensibility of allyl monomers to undergo hydrogen abstraction and the ability of plasma to abstract hydrogen atoms, including from allylic carbons by DBD plasma [38], might play a significant role. Otherwise, the detection of oligomers with hydrogen atoms as a terminal end group might reflect the occurrence of chain transfer, characteristic from the well-known allyl polymerization mechanism. Oligomers with the -CH_2_CH=CHOCH_2_(C_6_H_9_O_3_) end group could be related to the structure of the monomeric allyl radical, resulting from the hydrogen abstraction either by chain transfer reaction or by plasma-induced fragmentation. The contribution to the plasma polymerization of oxygen and water from the atmosphere, because of the open-air configuration of the plasma reactor, could be the origin of the detection of hydroxyl terminated oligomers. The relative concentration of each of these oligomeric structures to the A6CC monomer are plotted in Figure 9 for each polymerization time. As expected, and in agreement with NMR and GPC results, the detection of oligomeric species increases with the increase in plasma exposition time. The distribution of their structures does not seem to be affected by the number of runs under plasma. The abundance of [H-(A6CC)_n_-H + H^+^/NH_4_^+^] oligomers is around 2.5 times higher than their [H-(A6CC)_n_-CH_2_CH=CHOCH_2_(C_6_H_9_O_3_) + H^+^/NH_4_^+^] counter parts. Otherwise, the incorporation of hydroxyl groups seems to increase with the plasma exposition time, i.e., longer periods exposed to the atmospheric air.

The HRMS spectra also revealed the presence of a wide variety of signals (especially at low *m*/*z*), non-related to those associated with the monomer and oligomers (Figure S9). In particular, the relative high intensity of the peak with the *m*/*z* value of 161.081 was addressed. As discussed previously, a byproduct was detected in the NMR analyses. The combination of both HRMS and NMR spectroscopy results allowed us to propose a chemical structure for this byproduct (R’ in Figure 9). The simplest mechanism for the formation of this product could be the deallylation of the allyl ether pendent group of the A6CC monomer. While this origin remains speculative, it allowed us to address other peaks of the HRMS spectra where the detected masses supported the presence of an open form of the cyclic carbonate [R-(A6CC)_n_-H + H^+^/NH_4_^+^] (where n = 1 or 2 (361.186 and 578.318 *m*/*z* H+ or 378.212 *m*/*z* NH4+)). The proposed chemical structure, shown in Figure 9, resembles the structure that will be obtained by ring-opening polymerization of the A6CC initiated by the byproduct R’. In such a scheme, the alcohol moiety will act as the initiator by reacting with the carbonate carbon, leading to the production of polycarbonate oligomers [39]. Nevertheless, even if this secondary polymerization mechanism could be further supported with additional work, the HRMS results reported here clearly indicate that the plasma-induced polymerization of the A6CC monomer followed a free-radical polymerization mechanism with a high retention of the pendent cyclic carbonate.

## 4. Conclusions

For the first time, the feasibility of the plasma-induced free-radical polymerization of an allyl-substituted cyclic carbonate (A6CC) was demonstrated. The reaction products from the exposure of A6CC liquid layers to atmospheric-pressure DBD plasma were extensively investigated regarding the preservation of the pendent cyclic carbonate moieties and the structure of the oligomers formed. FTIR spectra confirmed the reaction of carbon–carbon double bonds and the formation of alkyl chains. Those findings agreed with the NMR results that further demonstrated the preservation of a carbonate cyclic structure, which is characterized by the interlocked diastereotopic protons of the carbonate ring. Increasing the plasma exposure time did not affect the structure of the cyclic carbonate but increased monomer conversion. After 90 s, A6CC conversion already reached 82% and rose by 10% when the exposure time was tripled. GPC analysis revealed the formation of oligomers, which agreed with the well-known low tendency of allyl monomers to homopolymerize. These findings already demonstrate the ability of atmospheric-pressure plasma to induce polymerization of the A6CC monomer. The determination of the chemical structure of the formed oligomers was attempted by coupled GPC-HRMS analyses. For the series of samples studied, independently of the plasma exposure time, oligomeric species of up to seven repeating units were mainly detected, which agreed with a chain-growth polymerization mechanism and clearly differed from a plasma-state polymerization. While the complexity of the HRMS spectra did not allow us to address all the masses detected, those with the higher intensity could be related to the formation of oligomers with three types of end groups, i.e., hydrogen atoms, hydroxyl and a derived form of the monomeric allyl radical of the A6CC. The role of hydrogen abstraction either by chain transfer or plasma-induced fragmentation was pointed out as a preliminary hypothesis. Additionally, the combination of the NMR and HRMS results allowed us to identify and propose a low-molecular-weight byproduct that was detected in all the studied samples. Tentatively, its origin was attributed to the plasma-induced deallylation of the A6CC monomer and its role as an initiator of a ring-opening polymerization of the cyclic carbonate introduced. Overall, the results presented in this work open the door to the room temperature and atmospheric-pressure copolymerization of allyl- and vinyl-substituted cyclic carbonates for the plasma deposition of functional thin films with pendent cyclic carbonates, for instance, to exploit the high reactivity of six-membered cyclic carbonates toward primary amines for sensing and biotechnological applications.

## Data Availability

The data presented in this study are available on request from the corresponding author.

## Supplementary Materials

The following are available online at https://www.mdpi.com/article/10.3390/polym13172856/s1, detailed assignment of the ^1^H NMR and FTIR spectra from the A6CC monomer. Figure S1: FTIR spectra of the samples after exposure of A6CC to different plasma powers and table runs. Figure S2: ^1^H NMR spectrum of the samples A6CC-90. Figure S3: COSY spectra of the series of samples. Figure S4: Chemical structures of the A6CC monomer polymerized by the allyl and the cyclic carbonate. Figure S5: HSQC-HMBC spectra of A6CC-40, -50 and -70. Figure S6: MW distribution of the series of samples collected by GPC-HRMS. Figure S7: Overlay representation of the GPC chromatogram and HRMS spectrum of the series of samples. Figure S8: potential structures by single plasma-induced homolytic cleavage of A6CC monomer. Figure S9: zoom of the A6CC-30 sample HRMS spectrum. Table S1: *m*/*z* values of the species monitored by high-resolution mass spectrometry.
