# Supplementary material for: Insights on the Atmospheric-Pressure Plasma-Induced Free-Radical Polymerization of Allyl Ether Cyclic Carbonate Liquid Layers
Source: Polymers (Basel). 2021 Aug 25;13(17):2856. doi: 10.3390/polym13172856 (PMC8434537; doi:10.3390/polym13172856)
Supplement: Supplementary file 1 [file polymers-13-02856-s001.zip › polymers-1348199-supplementary.pdf]

Supplementary Information

Insights on the Atmospheric-Pressure Plasma-Induced Free- Radical Polymerization of Allyl Ether Cyclic Carbonate Liquid Layers

Edyta M. Niemczyk[1,2], Alvaro Gomez-Lopez[3], Jean R.N. Haler[1], Gilles Frache[1], Haritz Sardon[3] and Robert Quintana[1,*]

[1] Department of Materials Research and Technology, Luxembourg Institute of Science and Technology (LIST), Belvaux, Luxembourg.
[2] University of Luxembourg, Esch-sur-Alzette, Luxembourg.
[3] POLYMAT and Polymer Science and Technology Department, Faculty of Chemistry, University of the Basque Country UPV/EHU, Paseo Manuel de Lardizabal 3, Donostia-San, Sebastián, 20018, Spain.

Complementary information of A6CC monomer chemical characterization.

FTIR (cm^{-1}): 2978 and 2954 (C-H stretching), 2911 (C–H asymmetric stretching), 2824 (C–H symmetric stretching), 1755 (C=O stretching from the carbonate), 1644 (C=C stretching), 1468 (C-H bending alkane), 1264 (C-O vibrational stretching), 1238 (O−C−O stretch), 1193 – 1050 (C-O-C stretching), 1030 (C-O-C ring skeletal vibration), 997 and 927 (C=C-H out of plane bending), 843 (out-of-plane bending mode of the carbonate O−C−O−O).
^{1}H NMR ($CDCl_3$, 60 0 MHz, 298 K) δ (ppm) = (assignment, coupling constant). δ = 0.91 (t, 3H, CH_2-CH_3, g, J = 7.6 Hz), 1.52 (q, 2H, CH_2-CH_3, f, ,J = 7.6 Hz), 3.39 (s, 2H, O-CH_2-C, e), 3.96 (d, 2H, CH_2=CH-CH_2-O, J = 5.6 Hz), 4.12 (d, 2H, C-CH_2-O-C(O)-O, J = 11.0 Hz), 4.32 (d, 2H, C-CH_2-O-C(O)-O, J = 11.0 Hz), 5.21 (q, 2H, CH_2=CH-CH_2-O, J = 18.0 Hz), 5.84 (m, 1H, CH_2=CH-CH_2-O, J = 6.2 Hz).

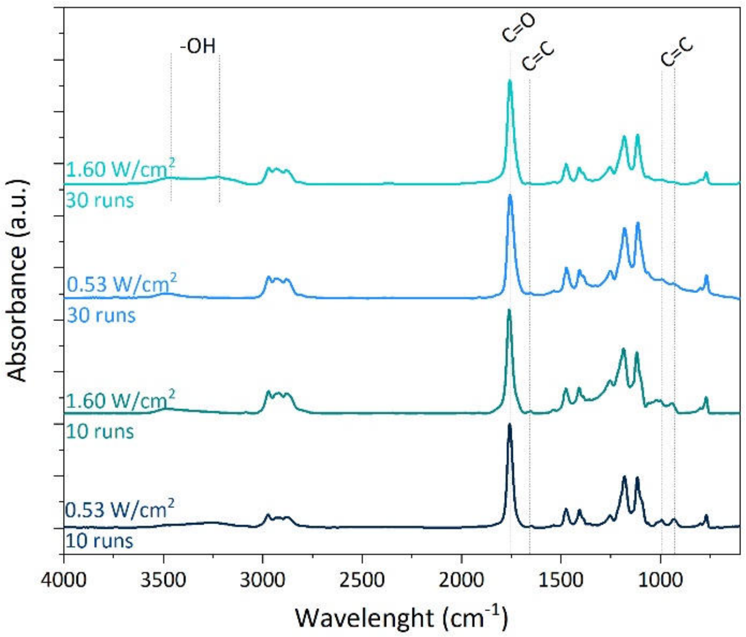

Figure S1. FTIR spectra of the samples after exposure of A6CC to different plasma power (0.53 and 1.60 W/cm^2) for 10 and 30 table runs.

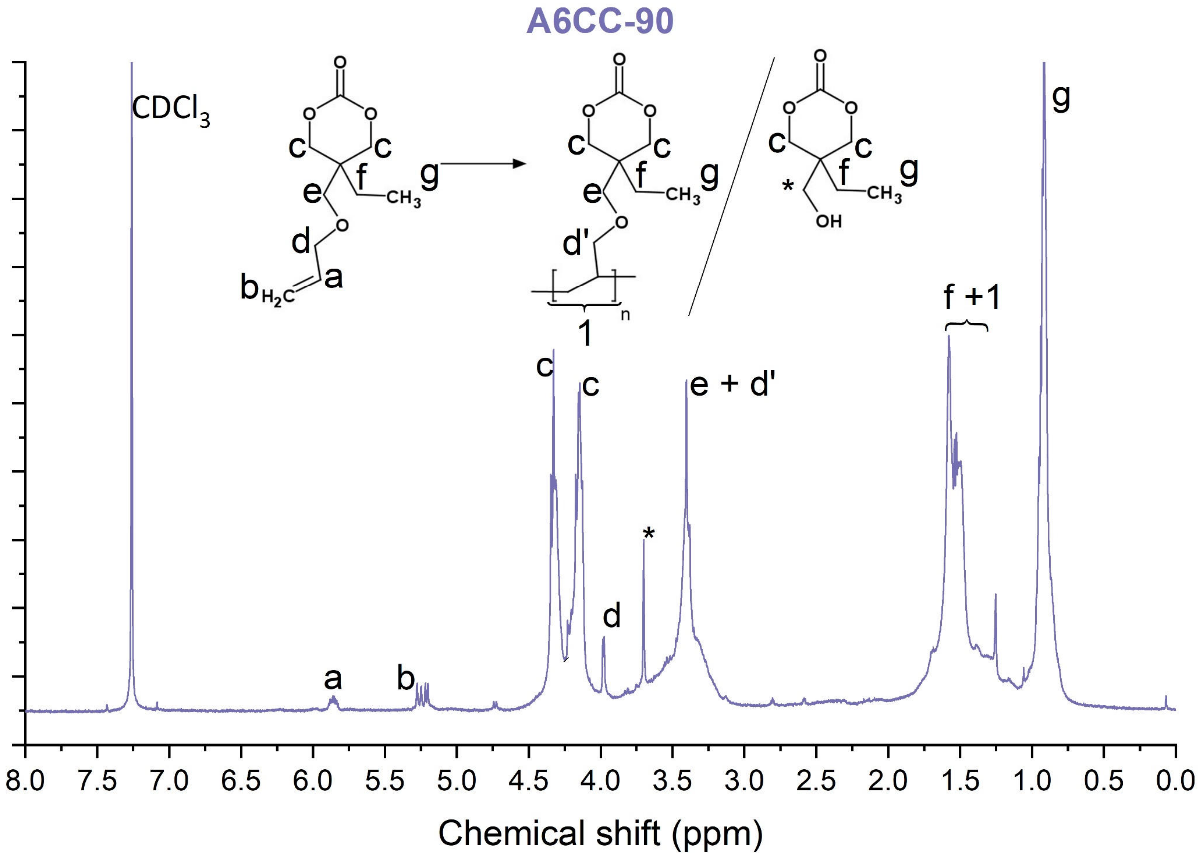

Figure S2. ^{1}H NMR spectrum of the samples A6CC-90 in $CDCl_3$.

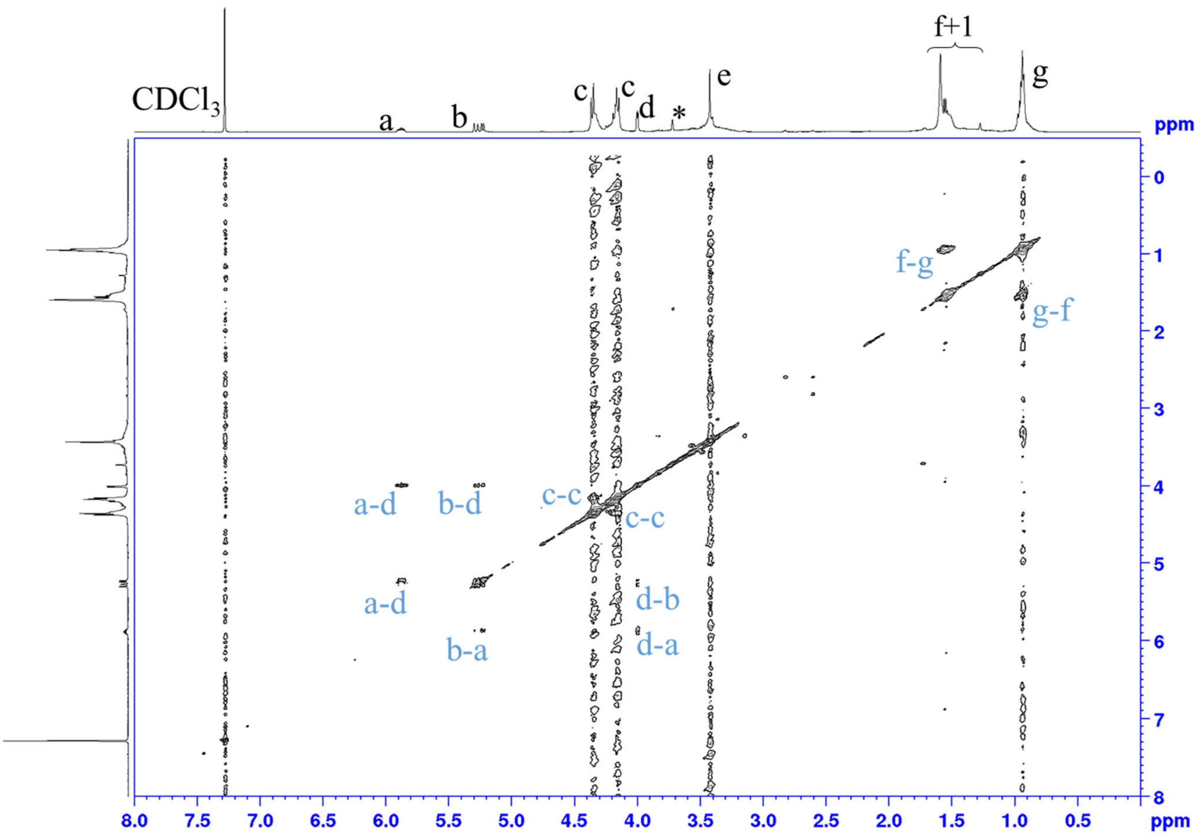

Figure S3a. COSY spectra of the A6CC-30. (*) corresponds to the methylene groups of the detected by-product.

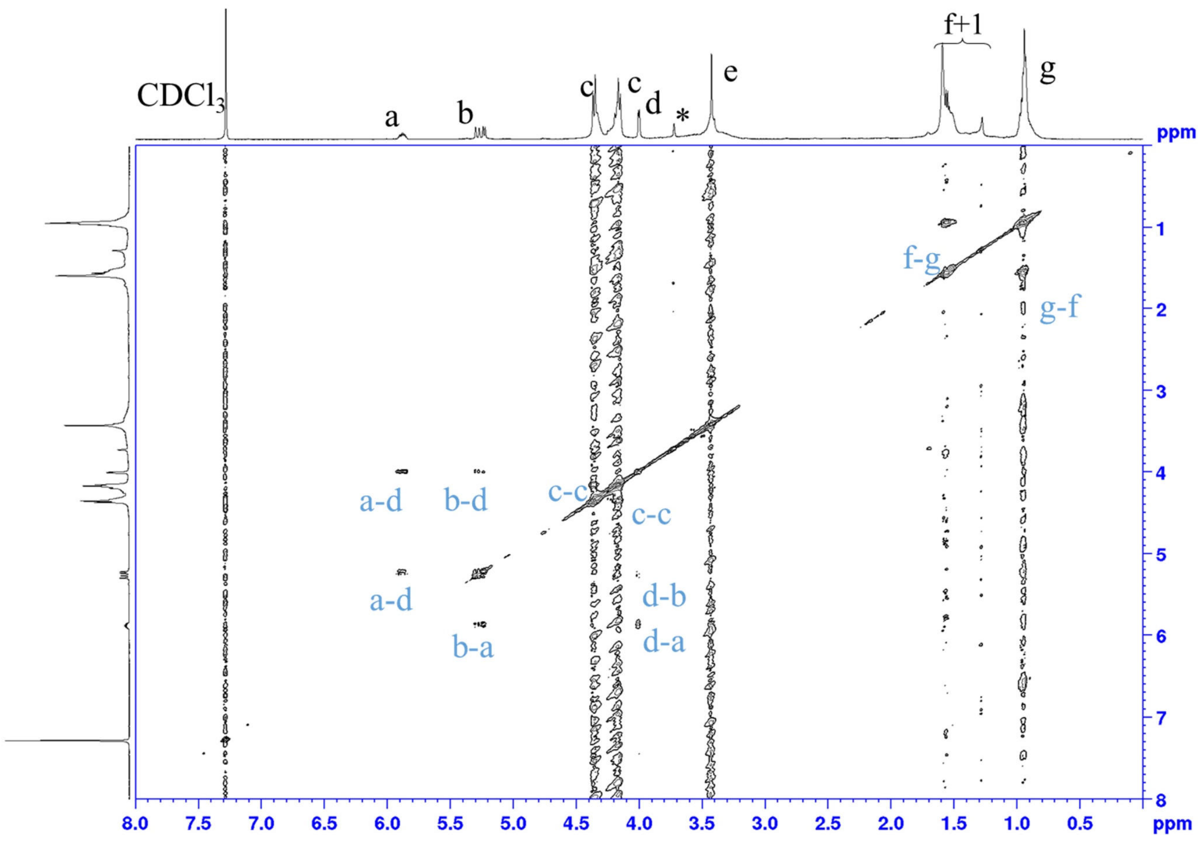

Figure S3b. COSY spectra of the A6CC-40. (*) corresponds to the methylene groups of the detected by-product.

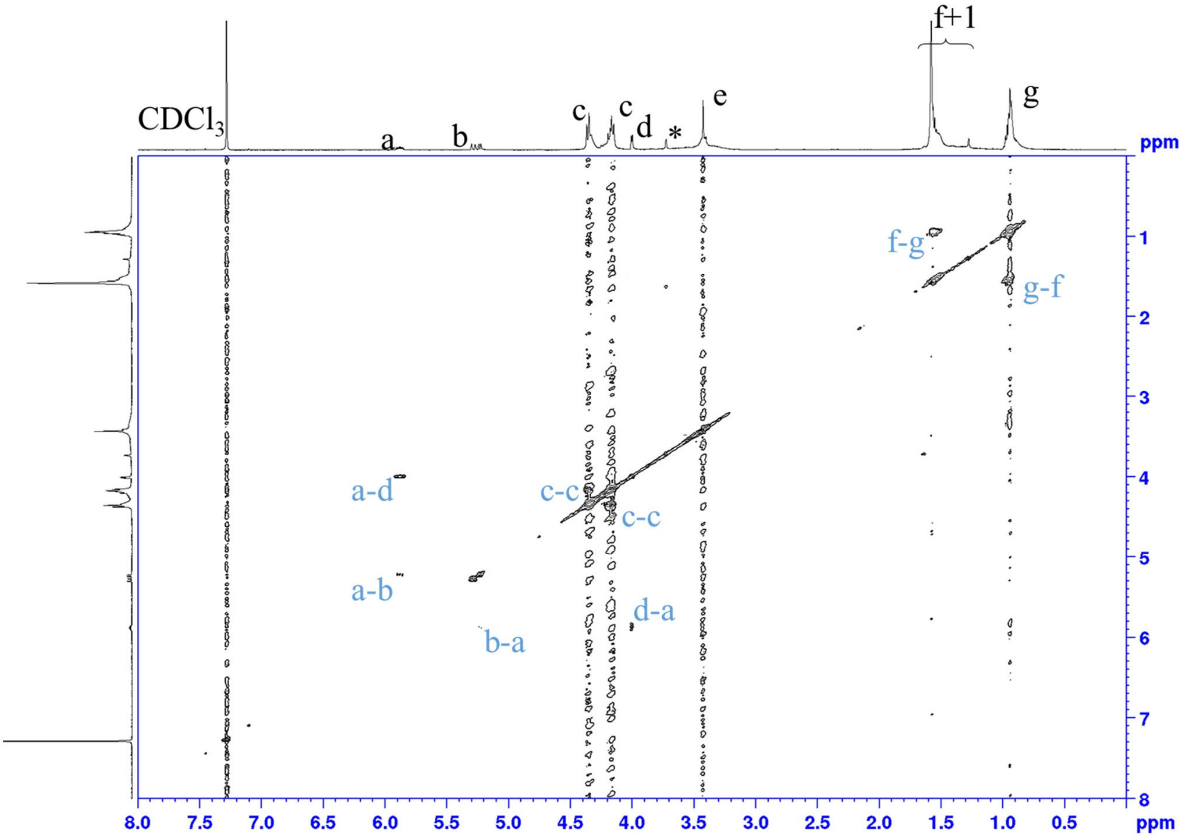

Figure S3c. COSY spectra of the A6CC-50. (*) corresponds to the methylene groups of the detected by-product.

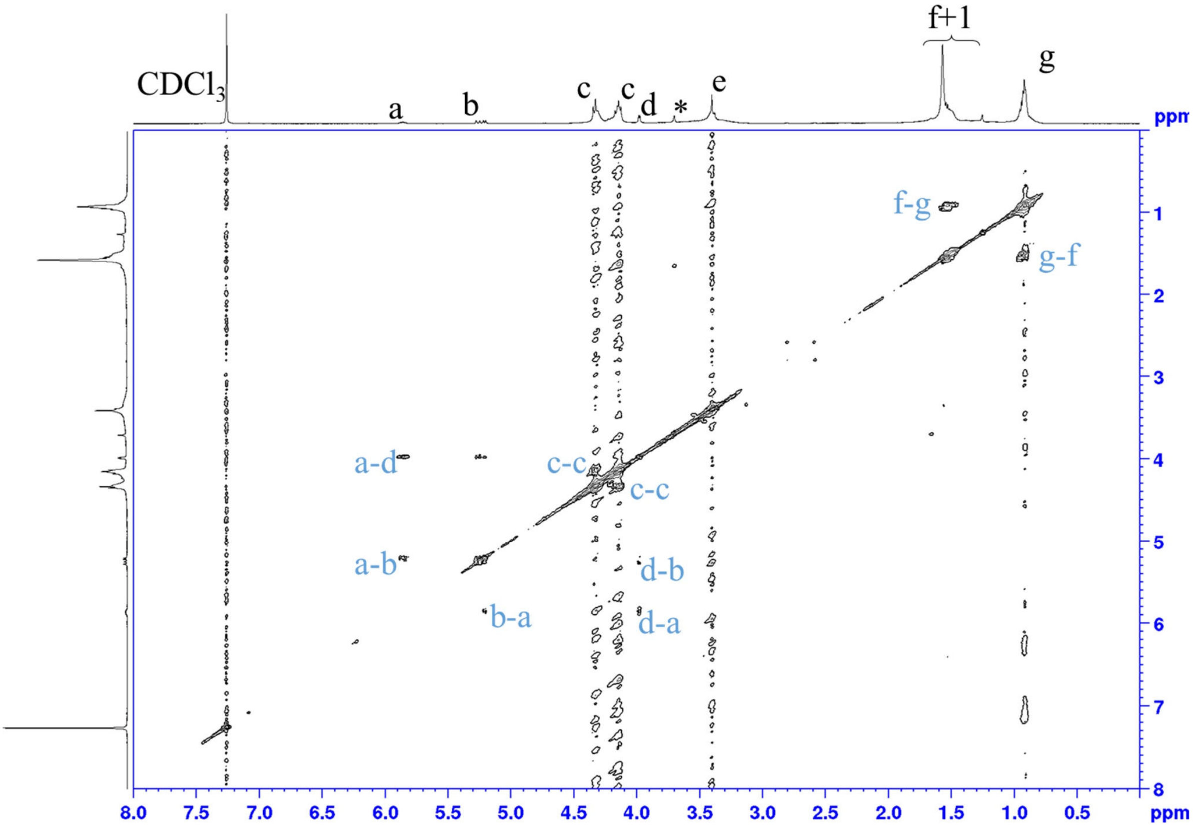

Figure S3d. COSY spectra of the A6CC-70. (*) corresponds to the methylene groups of the detected by-product.

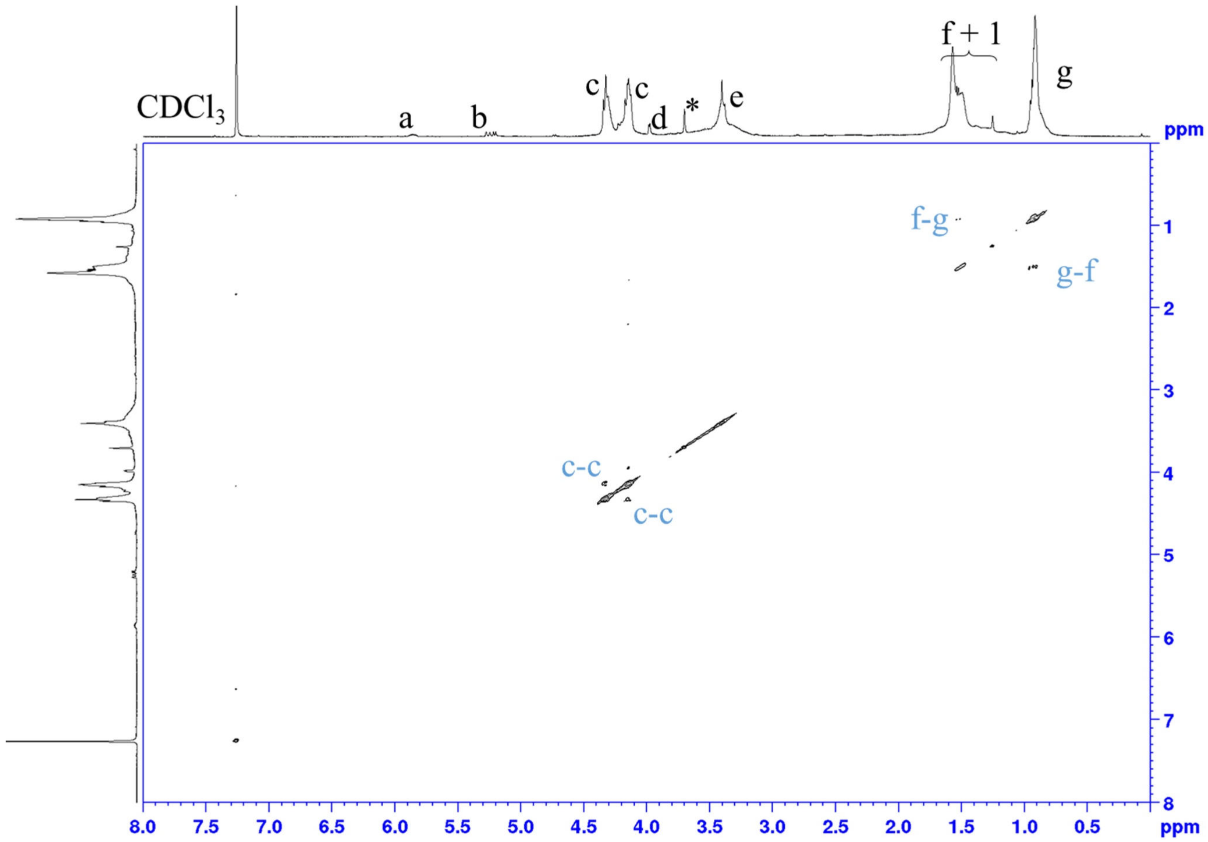

Figure S3e. COSY spectra of the A6CC-90. (*) corresponds to the methylene groups of the detected by-product.

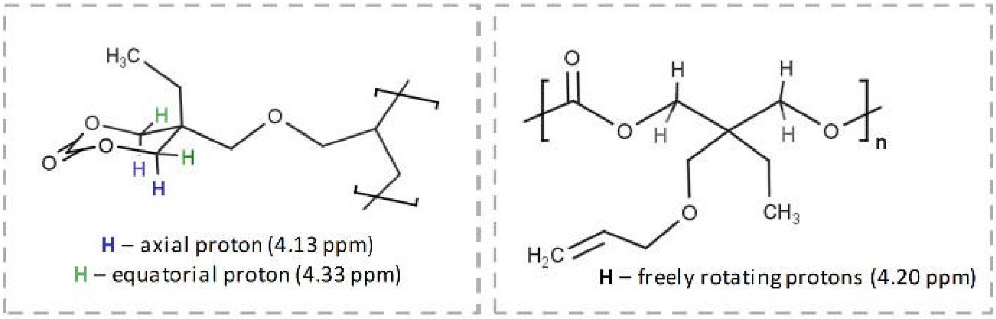

(**a**) (**b**)

Figure S4. Chemical structures of the A6CC monomer polymerized by the allyl bond (a) or by the cyclic carbonate (b) with indication of the chemical shifts of the protons from the methylene groups.

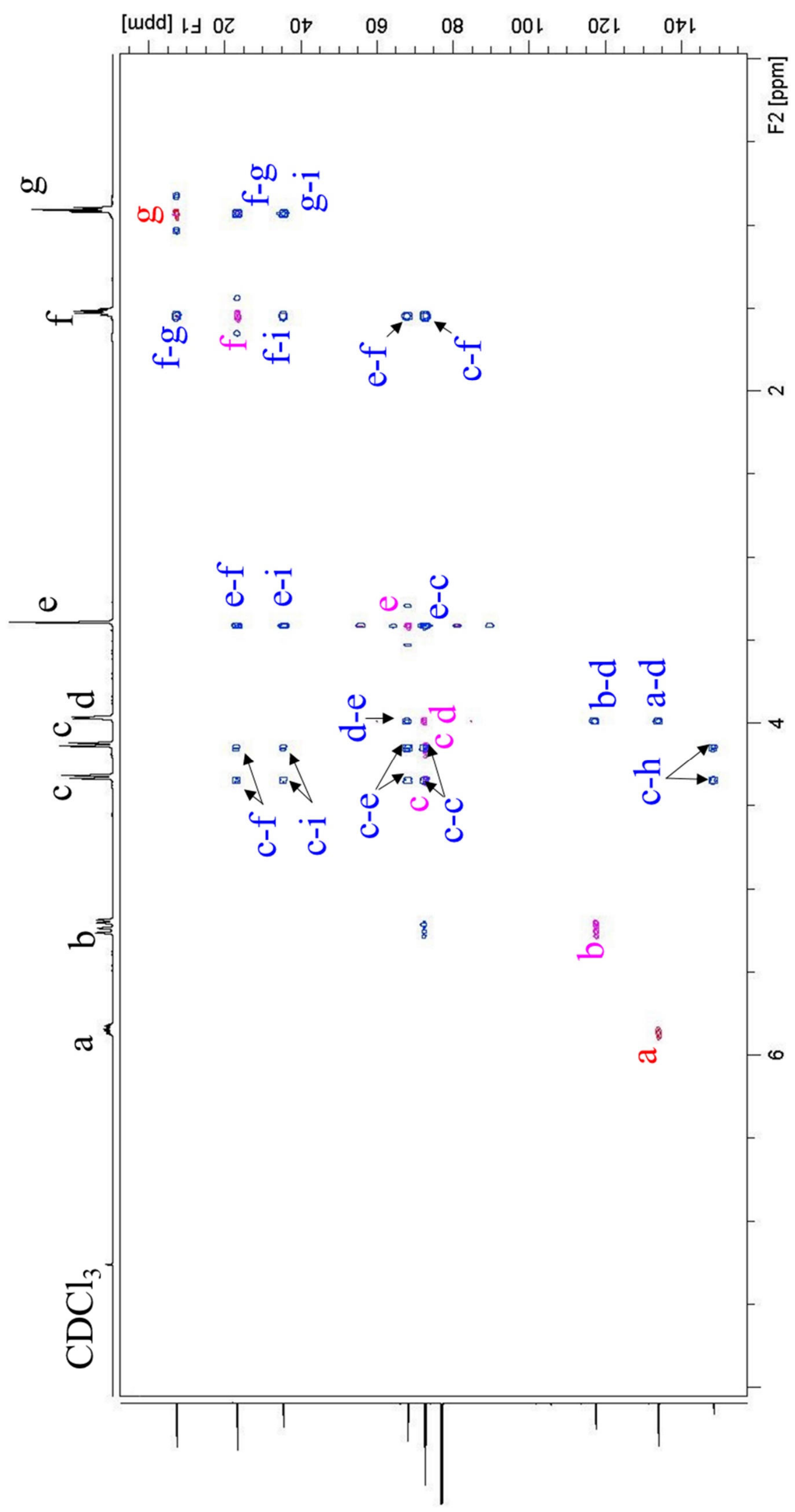

Figure S5a. HSQC-HMBC spectra of the A6CC monomer.

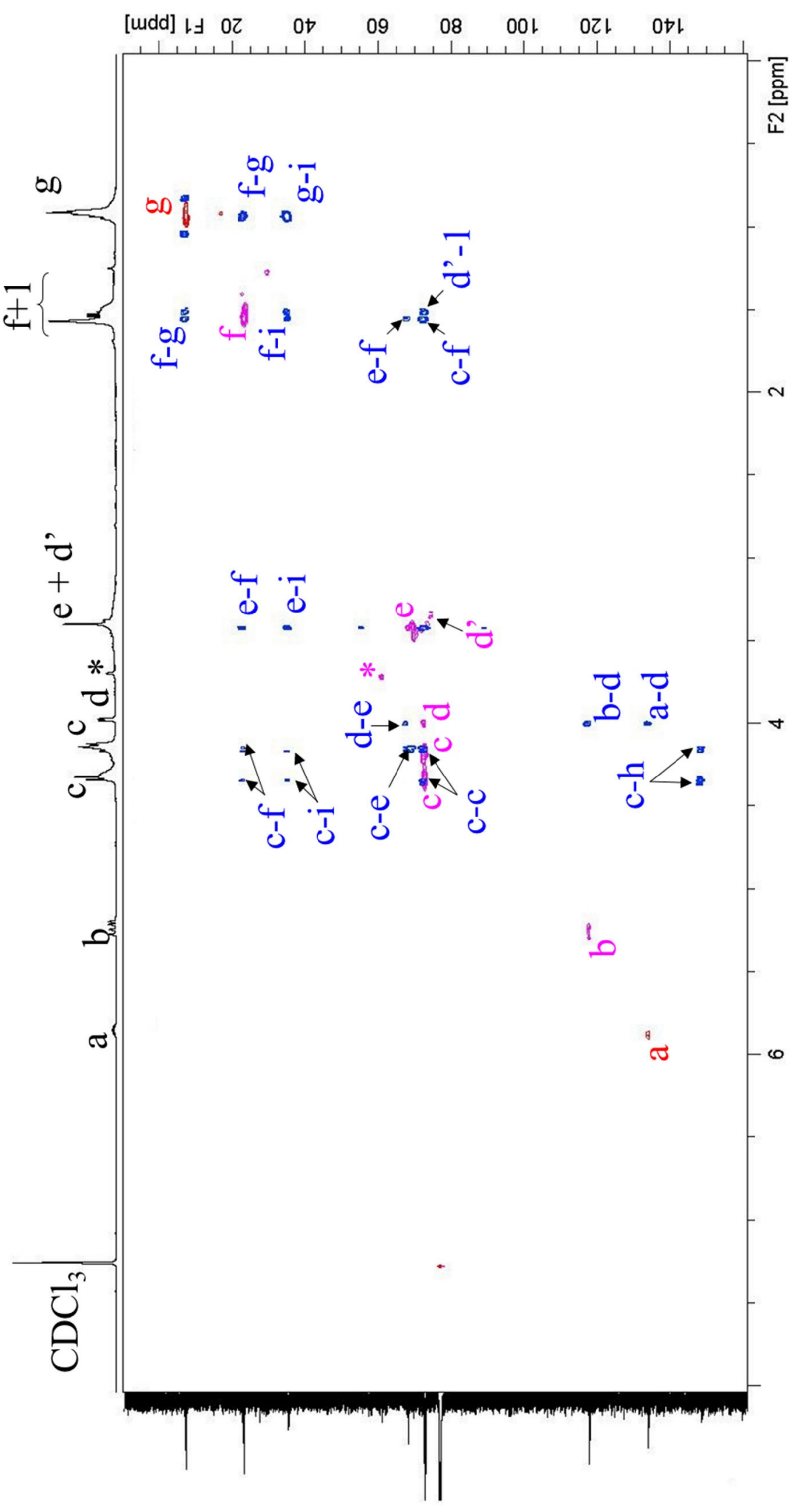

Figure S5b. HSQC-HMBC spectra of the A6CC-30. (*) corresponds to the methylene groups of the detected by-product.

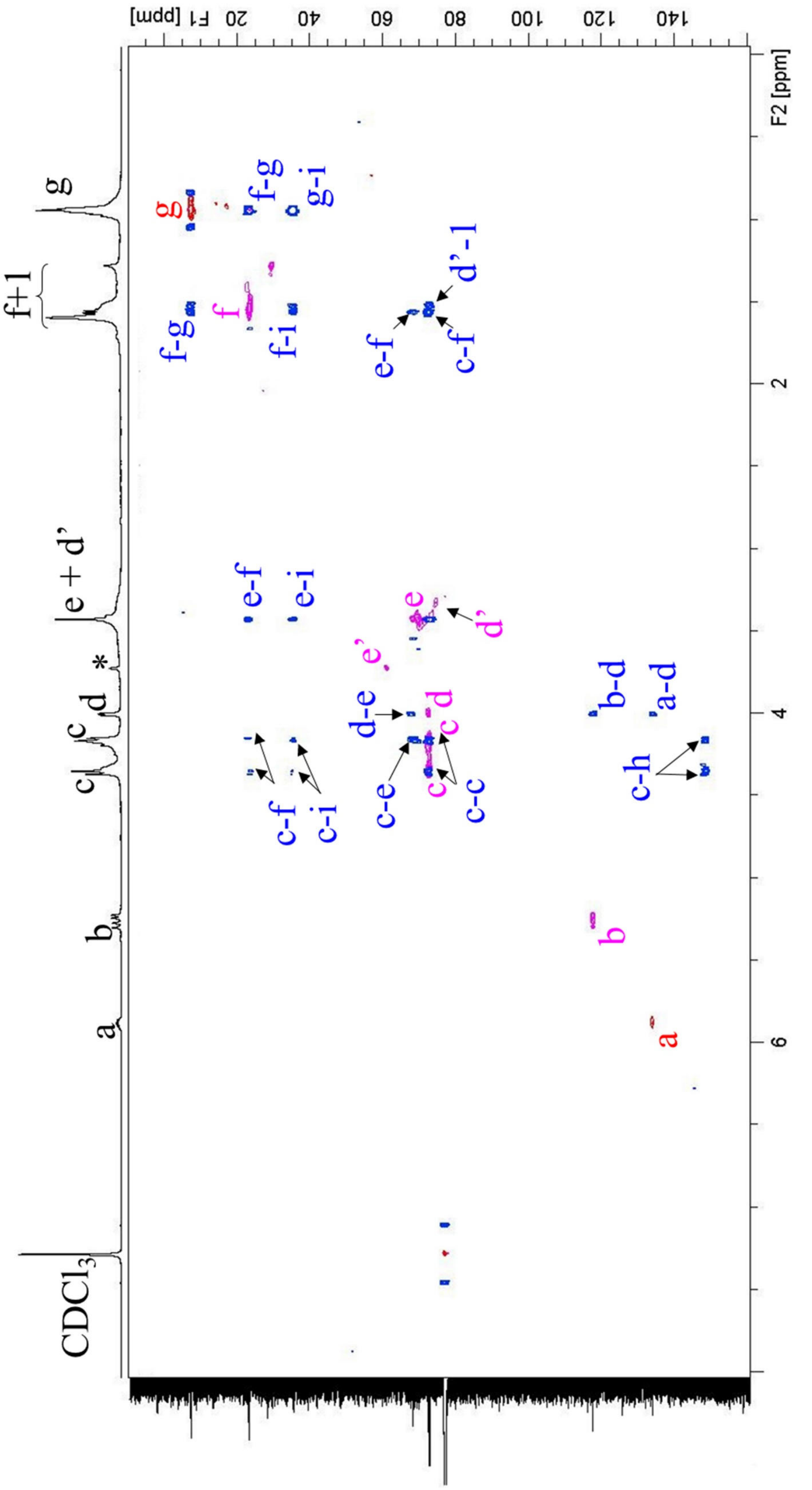

Figure S5c. HSQC-HMBC spectra of the A6CC-40. (*) corresponds to the methylene groups of the detected by-product.

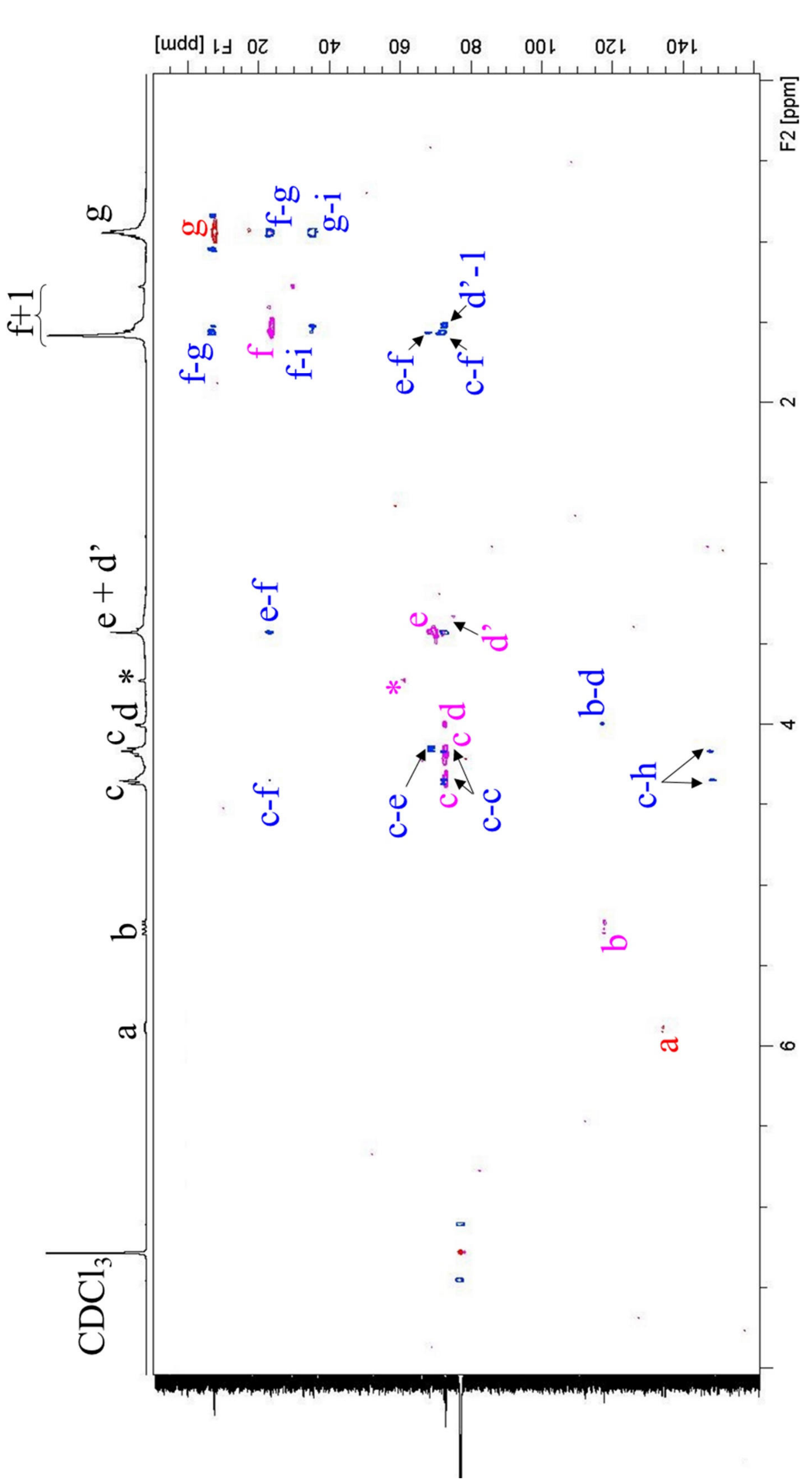

Figure S5d. HSQC-HMBC spectra of the A6CC-50. (*) corresponds to the methylene groups of the detected by-product.

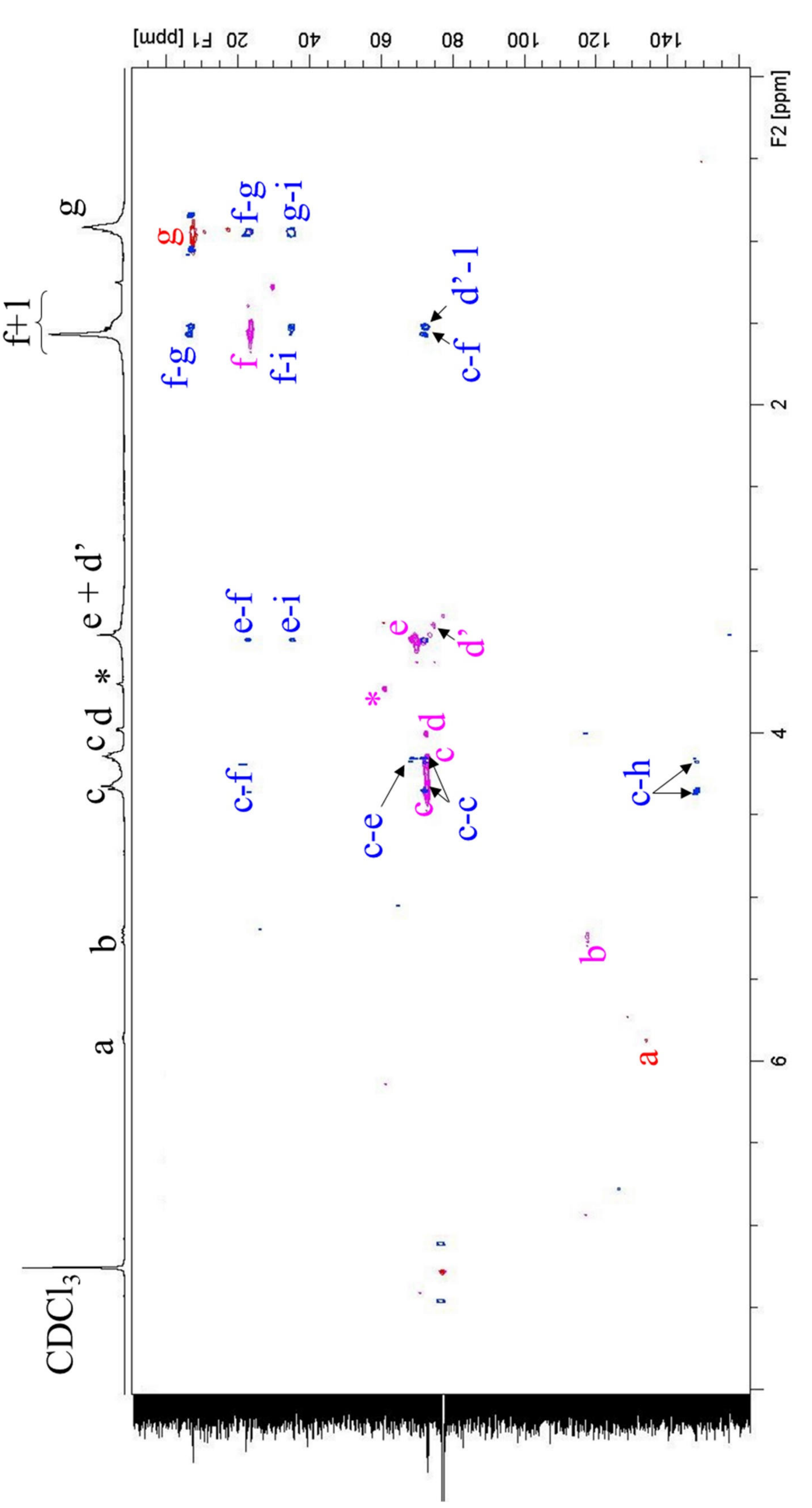

Figure S5e. HSQC-HMBC spectra of the A6CC-70. (*) corresponds to the methylene groups of the detected by-product.

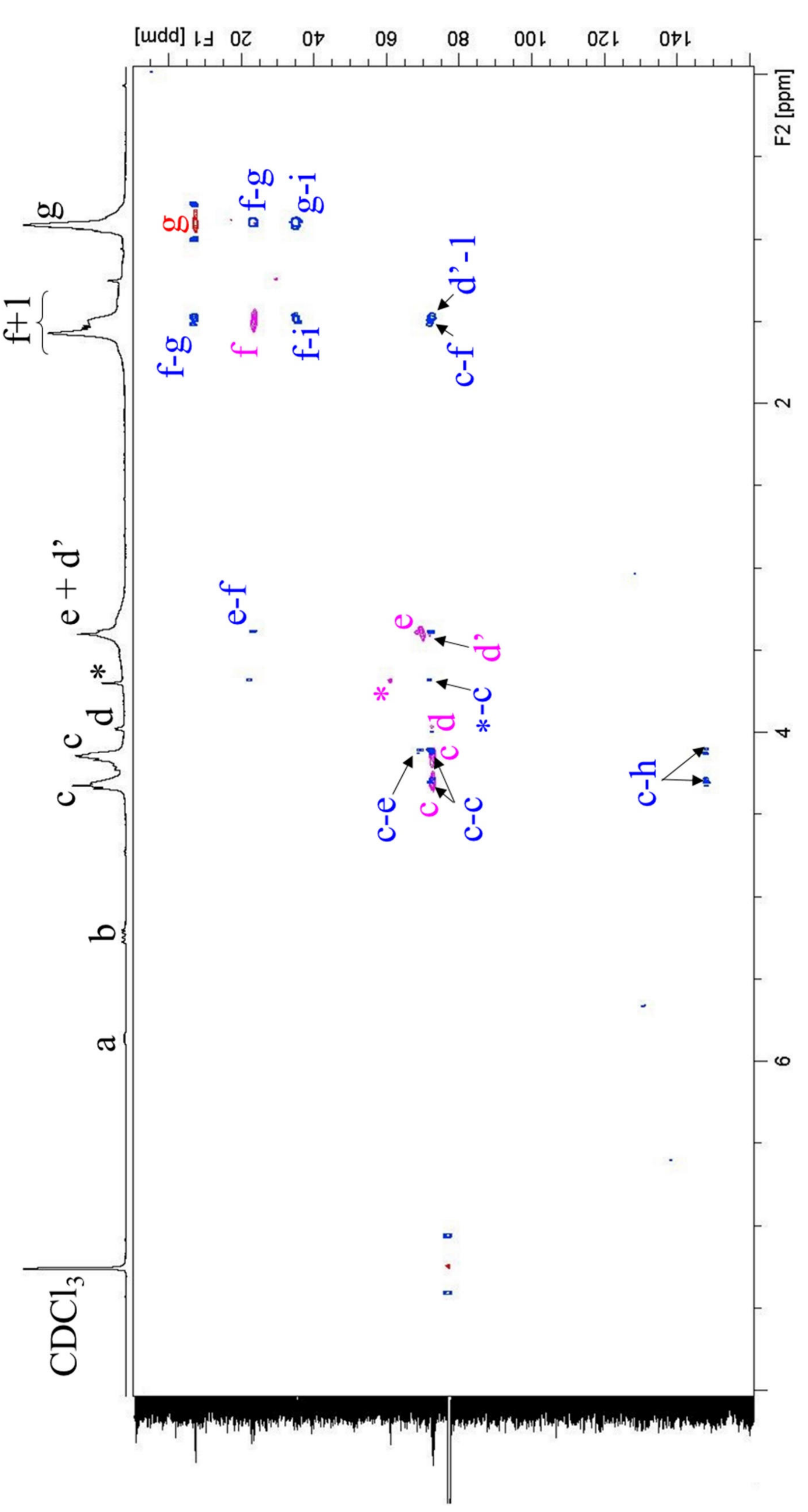

Figure S5f. HSQC-HMBC spectra of the A6CC-90. (*) corresponds to the methylene groups of the detected by-product.

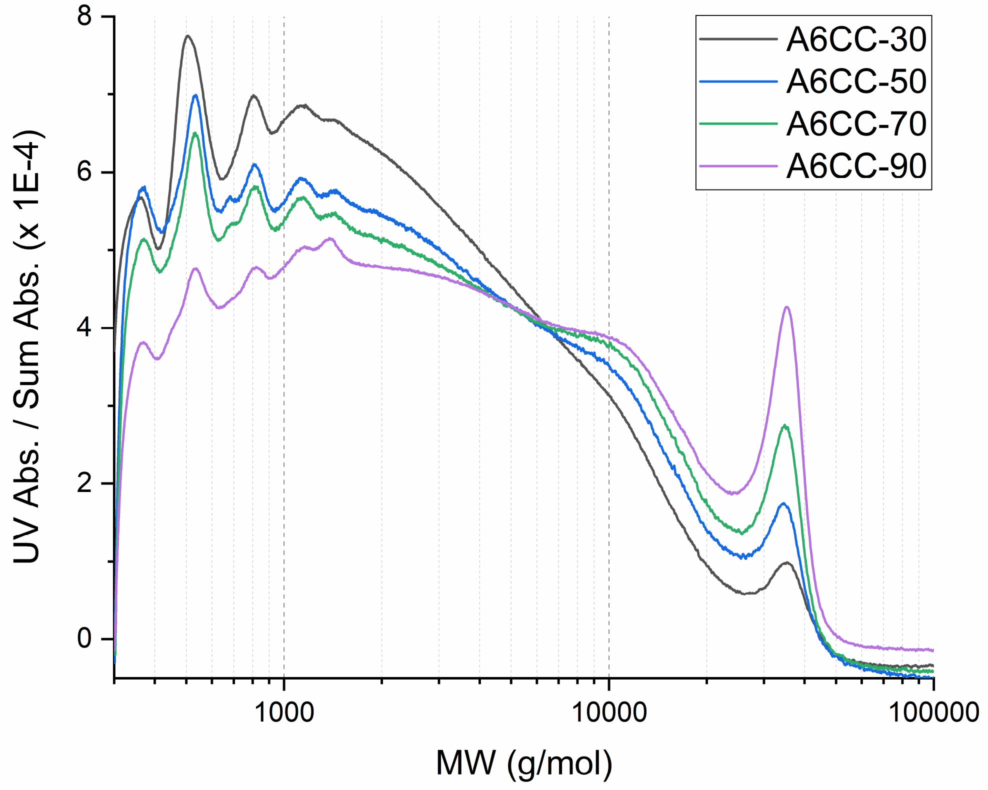

Figure S6. MW distribution of A6CC samples collected by GPC-HRMS using a mesopore column with an exclusion limit of 25k g·mol^{-1}.

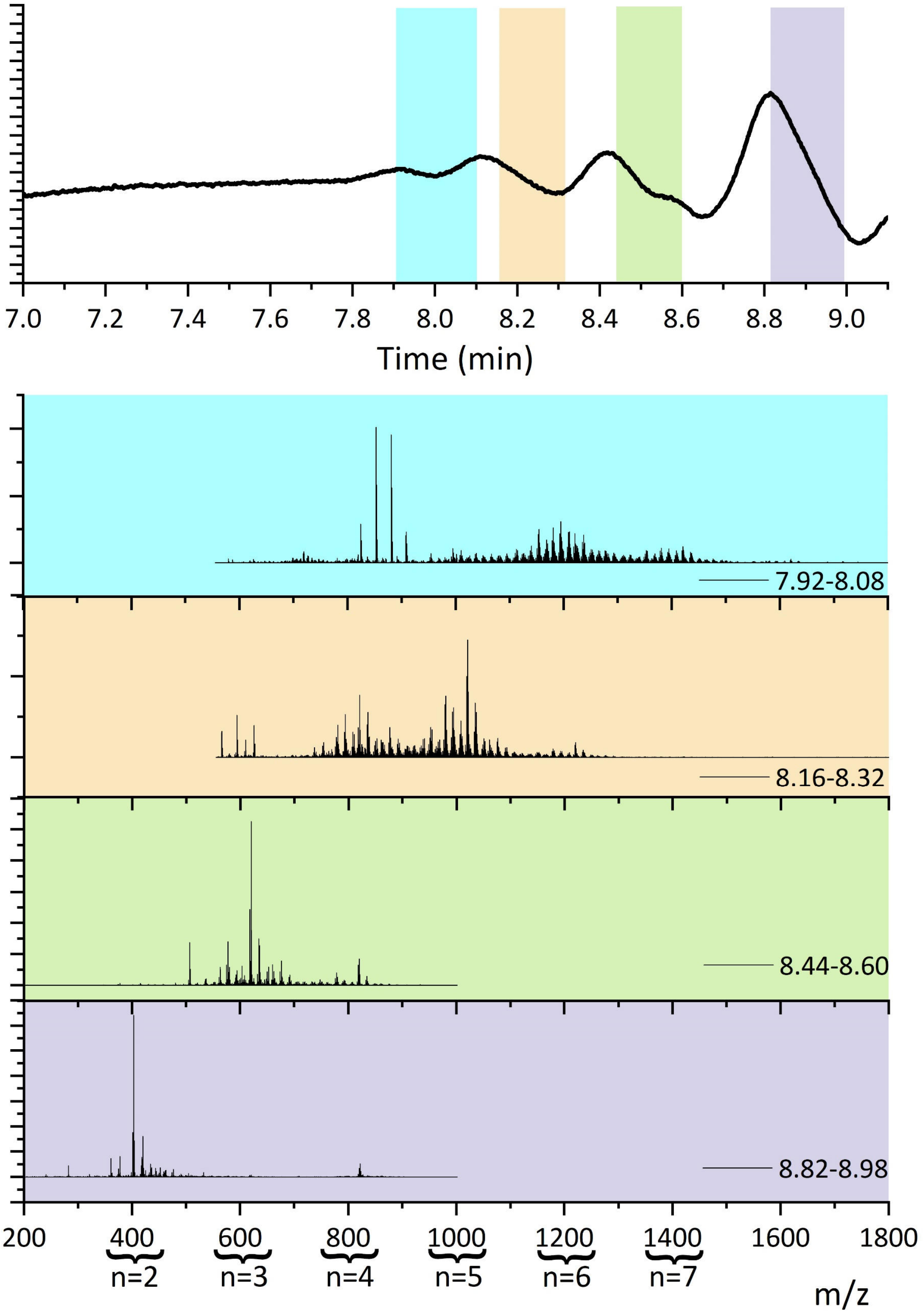

Figure S7a. Overlay representation of the GPC chromatogram and HRMS spectrum of A6CC-30. For each elution time range, a) 7.92 – 8.08 min, b) 8.16 – 8.32 min, c) 8.44 – 8.60 min and d) 8.82 – 8.98 min the corresponding HRMS spectrum is displayed. The ranges are indicated by color bands in the GPC chromatogram. For reference, the number of repeating units (n) of the detected oligomers is given.

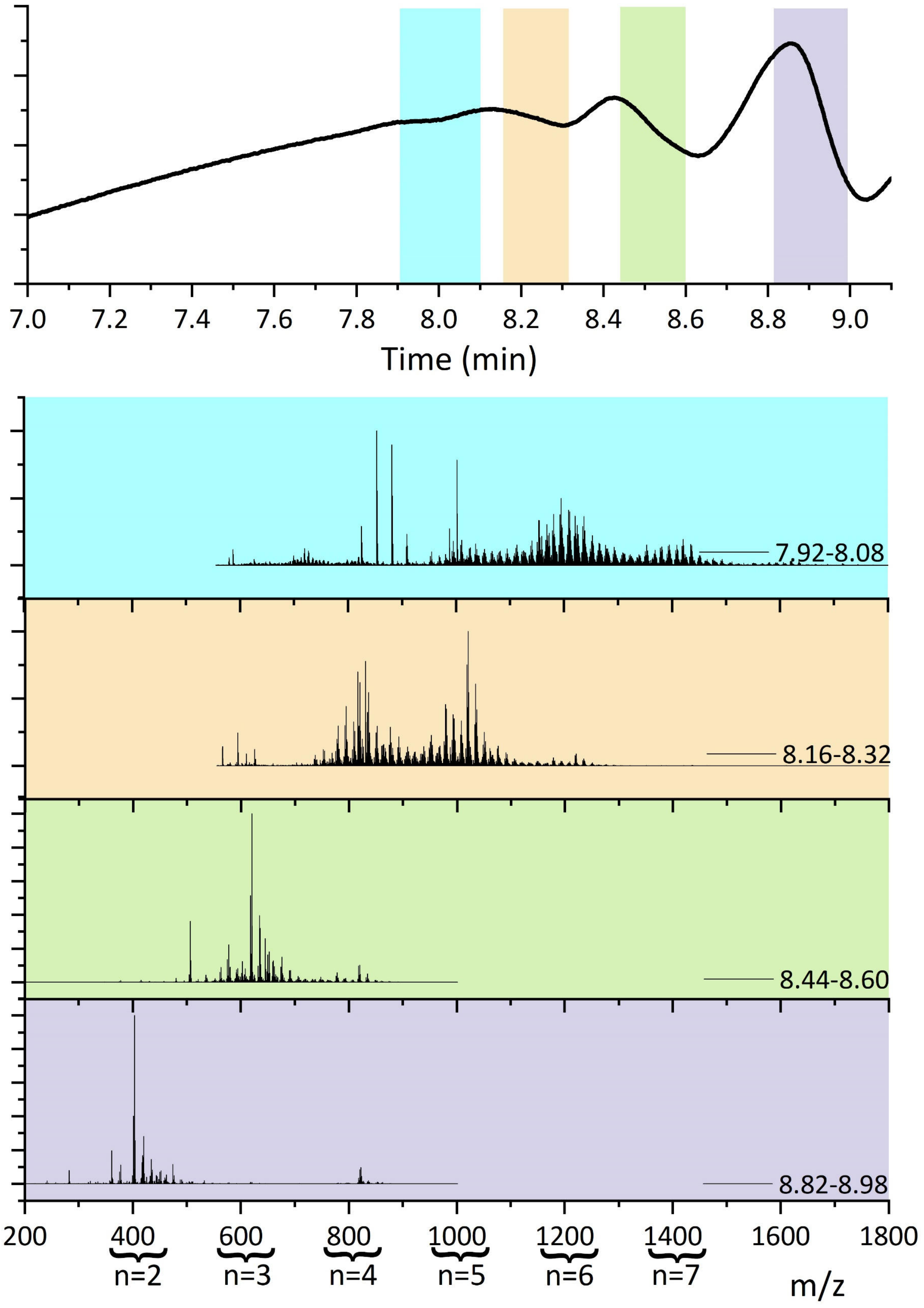

Figure S7b. Overlay representation of the GPC chromatogram and HRMS spectrum of A6CC-40. For each elution time range, a) 7.92 – 8.08 min, b) 8.16 – 8.32 min, c) 8.44 – 8.60 min and d) 8.82 – 8.98 min the corresponding HRMS spectrum is displayed. The ranges are indicated by color bands in the GPC chromatogram. For reference, the number of repeating units (n) of the detected oligomers is given.

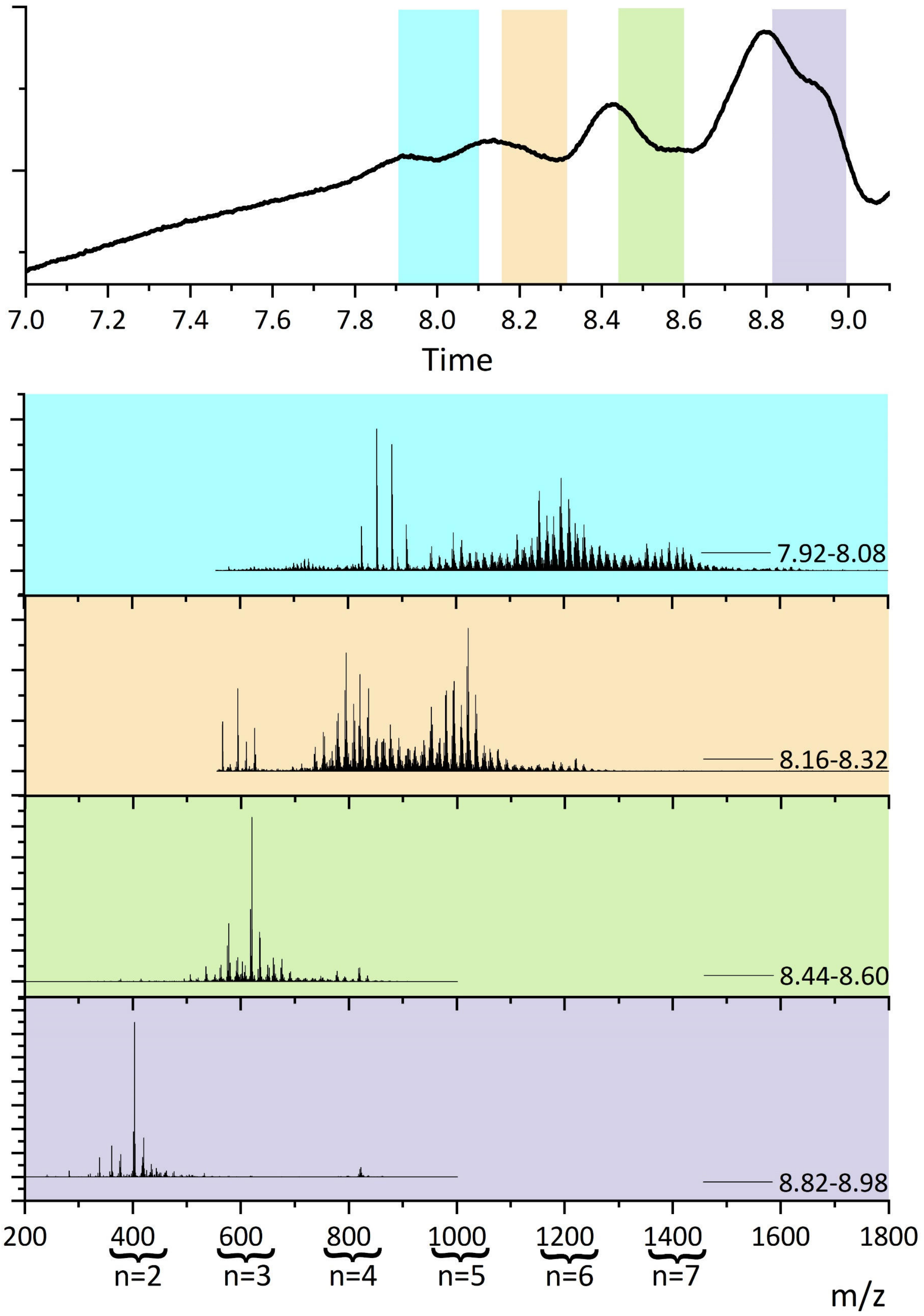

Figure S7c. Overlay representation of the GPC chromatogram and HRMS spectrum of A6CC-50. For each elution time range, a) 7.92 – 8.08 min, b) 8.16 – 8.32 min, c) 8.44 – 8.60 min and d) 8.82 – 8.98 min the corresponding HRMS spectrum is displayed. The ranges are indicated by color bands in the GPC chromatogram. For reference, the number of repeating units (n) of the detected oligomers is given.

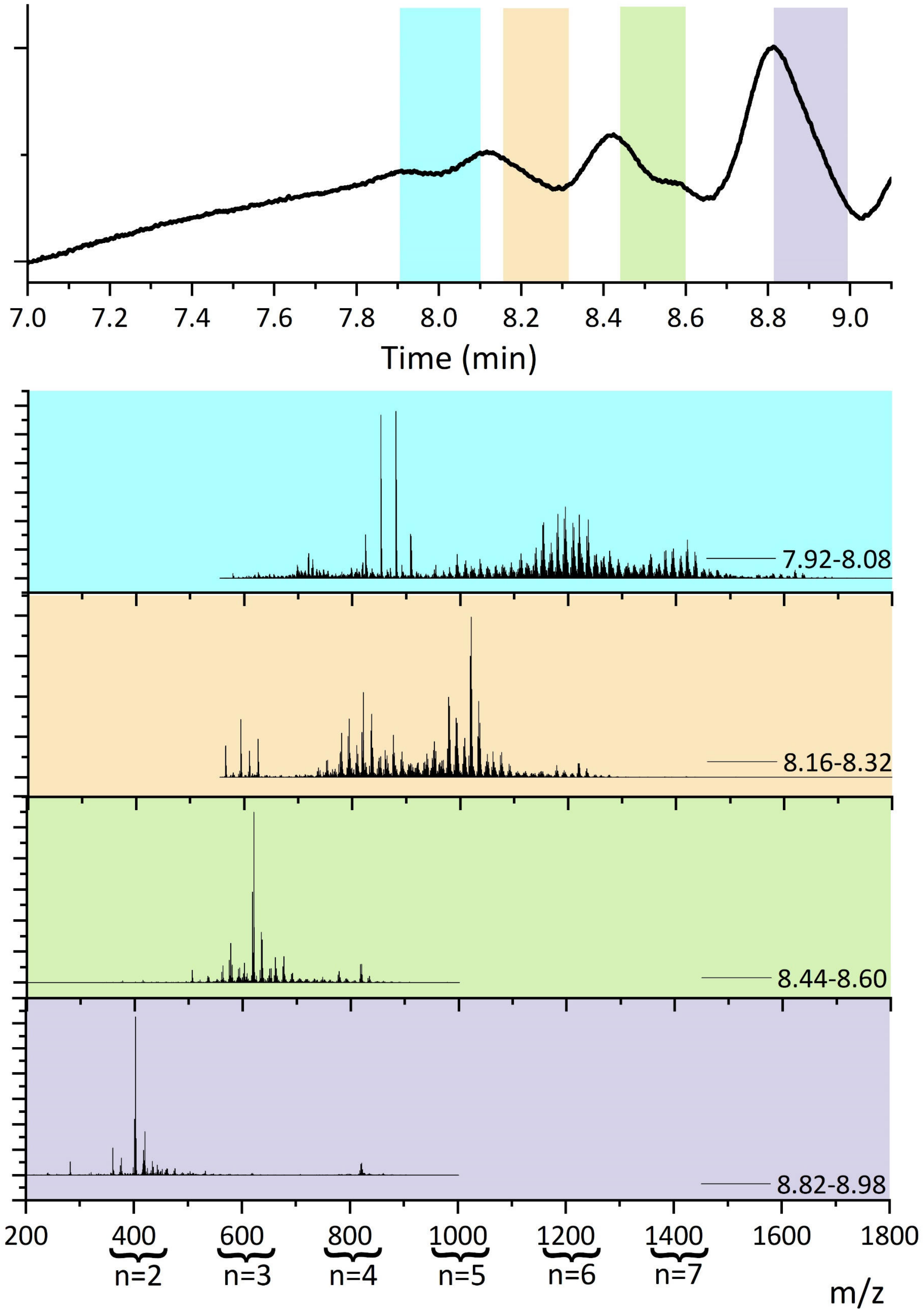

Figure S7d. Overlay representation of the GPC chromatogram and HRMS spectrum of A6CC-70. For each elution time range, a) 7.92 – 8.08 min, b) 8.16 – 8.32 min, c) 8.44 – 8.60 min and d) 8.82 – 8.98 min the corresponding HRMS spectrum is displayed. The ranges are indicated by color bands in the GPC chromatogram. For reference, the number of repeating units (n) of the detected oligomers is given.

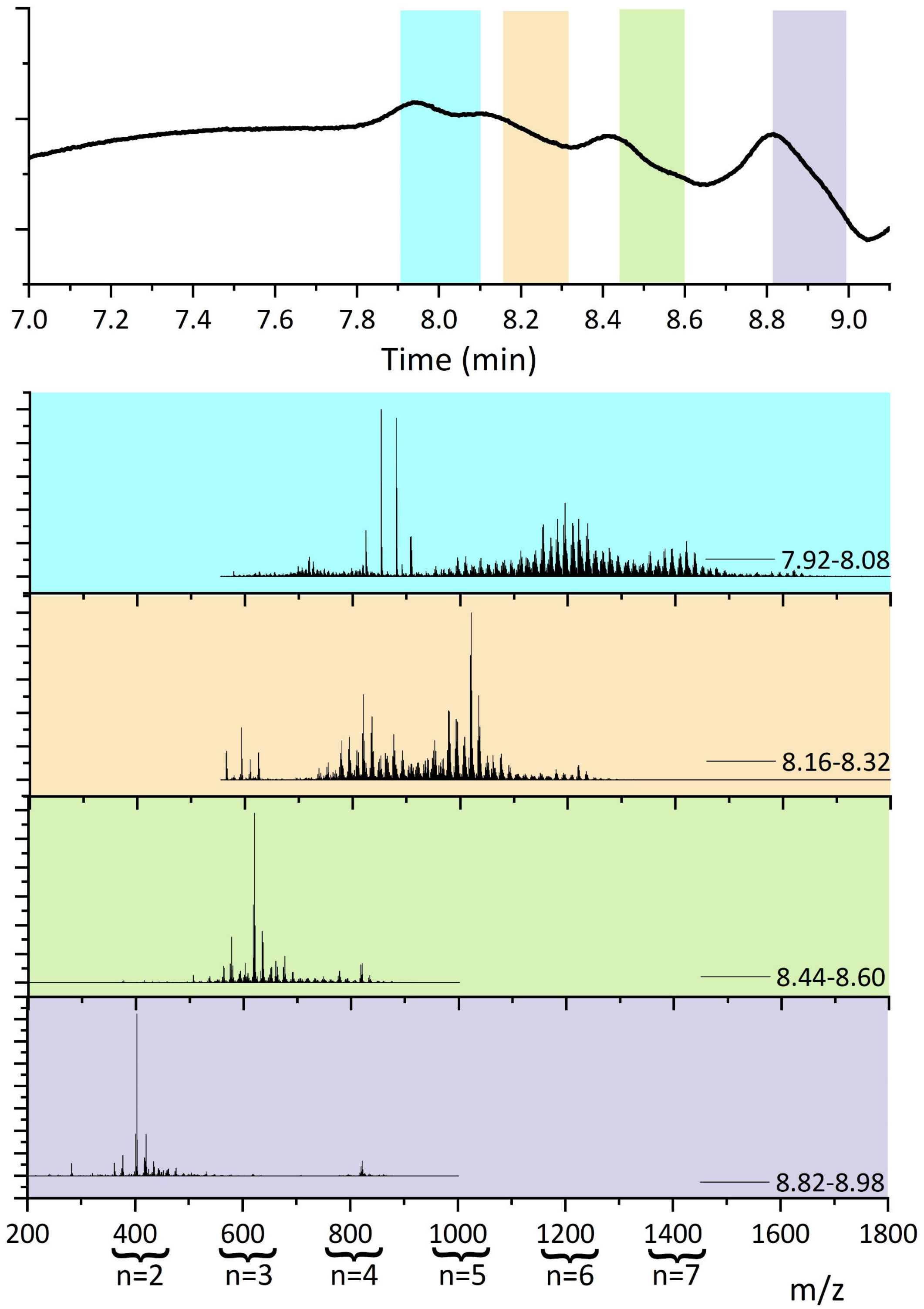

Figure S7e. Overlay representation of the GPC chromatogram and HRMS spectrum of A6CC-90. For each elution time range, a) 7.92 – 8.08 min, b) 8.16 – 8.32 min, c) 8.44 – 8.60 min and d) 8.82 – 8.98 min the corresponding HRMS spectrum is displayed. The ranges are indicated by color bands in the GPC chromatogram. For reference, the number of repeating units (n) of the detected oligomers is given.

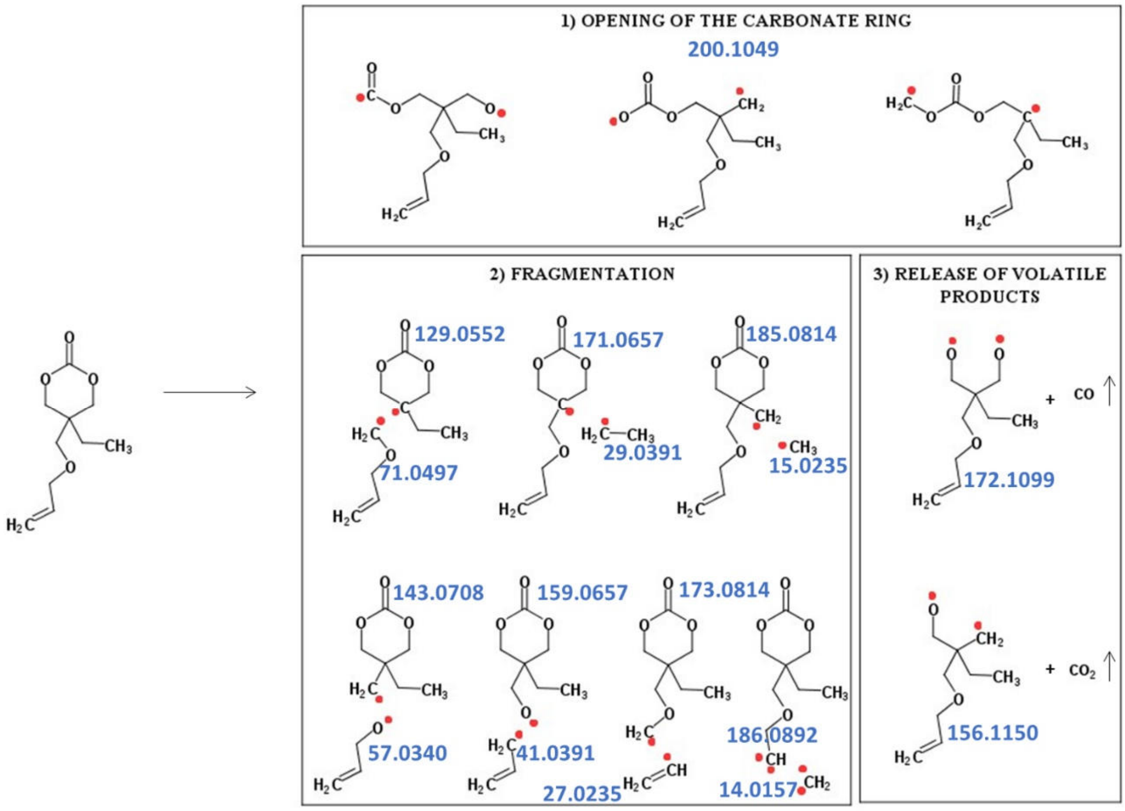

Figure S8. Chemical structures from a potential single plasma-induced homolytic cleavage of A6CC monomer, considering: 1) opening of the cyclic ring, 2) formation of two radicals and 3) release of volatile reaction products. In blue are given the exact mass of the fragments.

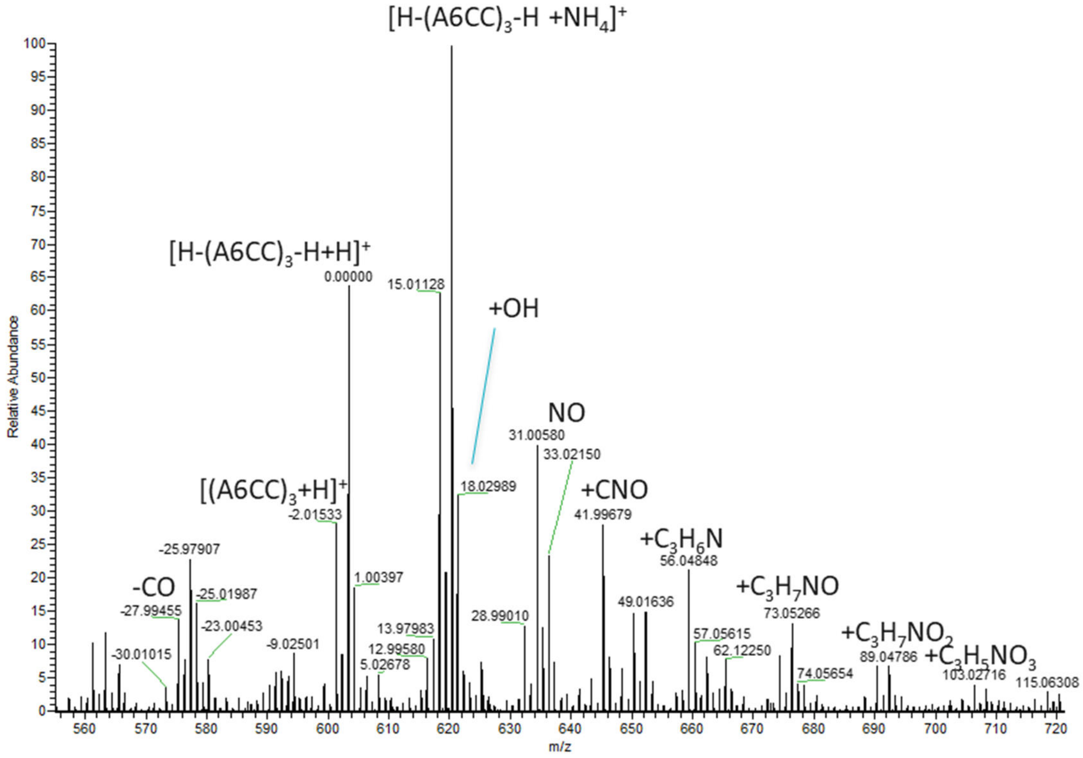

Figure S9. Zoom for A6CC-30 HRMS spectrum in the mass range related to A6CC trimer.

Table S1. List of relevant species monitored by High Resolution Mass Spectrometry (H+ and NH4+ as reagent ions) with their corresponding m/z values. R′ refers to structure shown in Figure 9. For each sample, the ratio refers to the relative signal intensities of the species in relation to the protonated monomer A6CC H+.

Structure	Reagent ion	m/z (-)	Ratio (-)				
			A6CC-30	A6CC-40	A6CC-50	A6CC-70	A6CC-90
(R')	H^+	161.081	0.225	0.212	0.240	0.310	0.571
**A6CC**	H^+	201.113	1.000	1.000	1.000	1.000	1.000
**A6CC**	NH_4^+	218.139	0.011	0.012	0.011	0.014	0.013
**OH-(A6CC)-H**	H^+	219.123	0.094	0.046	0.068	0.118	0.166
**OH-(A6CC)-H**	NH_4^+	236.149	0	0	0	0.005	0.007
**(R')-A6CC-H**	H^+	361.186	0.226	0.241	0.286	0.288	0.220
**(R')-A6CC-H**	NH_4^+	378.212	0.024	0.025	0.029	0.034	0.045
**H-(A6CC)$_2$-H**	H^+	403.233	0.585	0.538	0.824	1.190	1.618
**OH-(A6CC)$_2$-H**	H^+	419.228	0.107	0.067	0.092	0.131	0.176
**H-(A6CC)$_2$-H**	NH_4^+	420.260	0.179	0.162	0.238	0.339	0.475
**OH-(A6CC)$_2$-H**	NH_4^+	436.254	0.050	0.032	0.044	0.070	0.100
**(R')-A6CC$_2$-H**	NH_4^+	578.318	0.097	0.095	0.136	0.154	0.143
**H-(A6CC)$_3$-H**	NH_4^+	603.338	0.045	0.042	0.064	0.091	0.133
**OH-(A6CC)$_3$-H**	H^+	619.332	0.015	-	0.014	0.025	-
**H-(A6CC)$_3$-H**	NH_4^+	620.365	0.409	0.352	0.575	0.771	1.100
**OH-(A6CC)$_3$-H**	NH_4^+	636.359	0.095	0.071	0.105	0.140	0.196
**H-(A6CC)$_4$-H**	NH_4^+	820.469	0.234	0.176	0.298	0.387	0.571
**OH-(A6CC)$_4$-H**	NH_4^+	836.464	0.073	0.050	0.073	0.097	0.146
